# Therapy for Cancer: Strategy of Combining Anti-Angiogenic and Target Therapies

**DOI:** 10.3389/fcell.2017.00101

**Published:** 2017-12-07

**Authors:** Valentina Comunanza, Federico Bussolino

**Affiliations:** ^1^Department of Oncology, University of Torino, Candiolo, Italy; ^2^Candiolo Cancer Institute FPO-IRCCS, Candiolo, Italy

**Keywords:** cancer, VEGF, angiogenesis, target therapy, resistance

## Abstract

The concept that blood supply is required and necessary for cancer growth and spreading is intuitive and was firstly formalized by Judah Folkman in 1971, when he demonstrated that cancer cells release molecules able to promote the proliferation of endothelial cells and the formation of new vessels. This seminal result has initiated one of the most fascinating story of the medicine, which is offering a window of opportunity for cancer treatment based on the use of molecules inhibiting tumor angiogenesis and in particular vascular-endothelial growth factor (VEGF), which is the master gene in vasculature formation and is the commonest target of anti-angiogenic regimens. However, the clinical results are far from the remarkable successes obtained in pre-clinical models. The reasons of this discrepancy have been partially understood and well addressed in many reviews (Bergers and Hanahan, 2008; Bottsford-Miller et al., 2012; El-Kenawi and El-Remessy, 2013; Wang et al., 2015; Jayson et al., 2016). At present anti-angiogenic regimens are not used as single treatments but associated with standard chemotherapies. Based on emerging knowledge of the biology of VEGF, here we sustain the hypothesis of the efficacy of a dual approach based on targeting pro-angiogenic pathways and other druggable targets such as mutated oncogenes or the immune system.

## VEGF-targeted anti-angiogenic therapy

**Figure 1:**
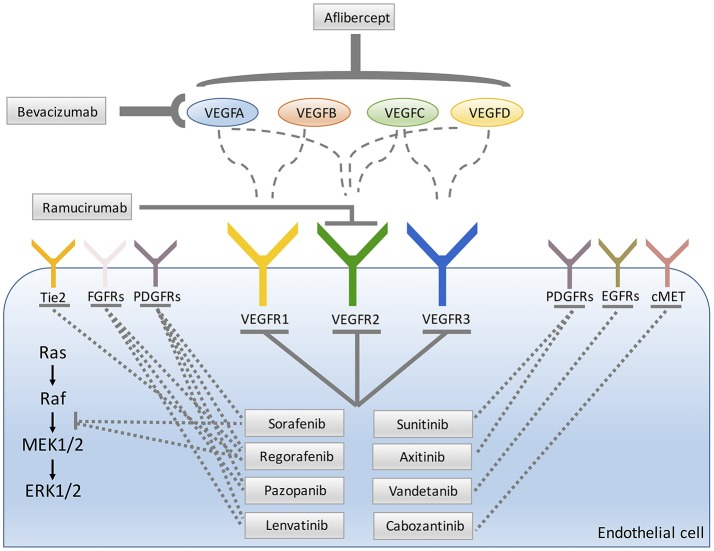
Main molecular targets of anti-angiogenic drugs approved for patients treatment.

During tumor progression, some clones experience the “angiogenic switch” by interrupting the balance between angiogenesis inducers and inhibitors and show pro-angiogenic phenotype. As a result, initial lesions or dormant metastases become more aggressive (Hanahan and Folkman, 1996; Wicki and Christofori, 2008). Angiogenesis inhibitors were postulated as anticancer drugs in the early 1970s (Folkman, 1971). Of all identified molecules that lead the blood vessel formation, VEGFA appears the main molecular driver of tumor angiogenesis. Indeed VEGFA is overexpressed in the majority of solid tumors and for this reason is the dominant target for antiangiogenic drugs (Carmeliet and Jain, 2000; Ferrara, 2002; Kerbel, 2008). VEGF/platelet-derived growth factor (PDGF) protein family is characterized by the presence of a structural motif with eight conserved cysteine residues forming the typical cystine-knot structure and include a wide range of angiogenic inducers: VEGFA, VEGFB, VEGFC, VEGFD, VEGFE, and placental growth factor (PLGF). The main signaling tyrosine kinase receptor (TKR) is VEGF-receptor, VEGFR2 (also known as KDR) (Ferrara and Kerbel, 2005). Two other VEGFRs are VEGFR1 and VEGFR3 (Figure 1). In embryo as well as in solid tumors VEGF expression is primarily stimulated by hypoxia and VEGFA transcription is promoted by hypoxia-inducible factor-1α (HIF1α) and −2α (HIF2α) that sense the reduced pO_2_ (Semenza, 2009).

Besides favoring the tumor feed, a consequence of “angiogenic switch” is the abnormality of the vessel architecture and defects of microcirculation rheology. The aberrant amounts of angiogenic inducers accelerate the proliferation of endothelial cells with a reduced time frame to allow the whole capillary maturation. As consequence, capillaries are tortuous, irregularly fenestrated with reduced pericyte coverage and leaky. These morphological aberrations induce the increase of interstitial pressure with the decrease of convective transport of small molecules including chemotherapeutics (Nagy et al., 2006; Jain, 2014).

The rationale proposed by Dr. Folkman to exploit anti-angiogenic compounds in clinical settings was to starve cancer and induce its dormancy. Currently, preclinical data suggest that a drastic pruning of tumor vasculature results in a selection of more aggressive cancer clones, which sustain disease progression (Ebos et al., 2009; Pàez-Ribes et al., 2009; Maione et al., 2012). Of interest, some studies failed to document such effects in other preclinical models (Singh et al., 2012).

However, before reaching the whole collapse of vascular bed, VEGF pathway blockade is characterized by an early and transient phase in which vessels assume normal shape and function (Folkman, 2006; Carmeliet and Jain, 2011; Goel et al., 2011). This normalization is characterized by rescue of the balance between inhibitors and inducers of angiogenesis, reduction of leakage and interstitial pressure, improvement of tumor perfusion and oxygenation, and drug delivery. This effect is potentially sensitizing for radiotherapy and increases tumor exposure to cytotoxic chemotherapy (Jain, 2005, 2014).

The degree of vascular normalization correlated with increased survival in glioblastoma multiforme (GBM) preclinical models (Kamoun et al., 2009) and patients (Sorensen et al., 2012; Batchelor et al., 2013). Vascular normalization can also improve trafficking of immune effector cells into tumor microenvironment and prolongs the survival of tumor-bearing mice receiving active immunotherapy (Huang et al., 2013; Jain, 2014; Kwilas et al., 2015).

### Anti-angiogenic regimens in advanced cancers

In general, the inhibition of tumor angiogenesis can be reached by the withdrawal of pro-angiogenic molecules or by inhibiting the signaling pathways triggered by these molecules. Most of angiogenesis inhibitors approved in human cancers targets VEGFA and its VEGFRs pro-angiogenic–mediated signals. The pioneer of angiogenesis inhibitors is the VEGFA-targeted monoclonal antibody bevacizumab (Ferrara et al., 2004; Kerbel, 2008; Figure 1). In contrast to most preclinical studies, monotherapy with bevacizumab failed to increase patients' overall survival (OS) (Jain, 2005), but in combination with chemotherapy it can extend progression free survival (PFS) and/or OS in several cancer types including metastatic colorectal cancer (mCRC) (Hurwitz et al., 2004; Giantonio et al., 2007; Cunningham et al., 2013) and recurrent GBM (Vredenburgh et al., 2007; Friedman et al., 2009). Based on the results of these trials, bevacizumab was approved for the treatment of patients with late stage CRC, non-small cell lung cancer (NSCLC), ovarian cancer, metastatic cervical cancer, metastatic renal cell carcinoma (RCC), and GBM, only when given in combination with chemotherapy (Table 1). As concern breast cancer, Food and Drug Administration (FDA) approved the combination of bevacizumab with paclitaxel for the treatment of human epidermal growth factor receptor 2 (HER2)-negative metastatic breast cancer (Miller et al., 2007). However, three further phase III trials, failed to confirm the efficacy of the association of bevacizumab with chemotherapy in metastatic breast cancer (Miles et al., 2010; Brufsky et al., 2011; Robert et al., 2011a) resulting in the withdrawal of approval by FDA.

**Table 1:** Approved VEGF-targeted therapy for oncology.

**Drug**	**Brand name**	**Mechanism**	**Indications**
Bevacizumab	Avastin (Genentech)	Monoclonal anti-VEGF antibody	CRC; NSCLC; RCC; GBM; epithelial ovarian cancer; fallopian tube cancer; primary peritoneal cancer; cervical cancer
Aflibercept	Zaltrap (Sanofi and Regeneron Pharmaceuticals)	Recombinant fusion VEGF protein	CRC
Ramucirumab	Cyramza (Eli Lilly and Company)	Monoclonal anti-VEGFR2 antibody	CRC; NSCLC; gastric or gastroesophageal junction adenocarcinoma
Sorafenib	Nexavar (Bayer)	Multi-TKI (VEGFRs, PDGFRs, RAF, KIT, FLT3, RET)	RCC, HCC, thyroid cancer
Sunitinib	Sutent (Pfizer)	Multi-TKI (VEGFRs, PDGFRs, FLT3, CSF1R, RET)	RCC, pancreatic neuroendocrine tumors, gastrointestinal stromal tumors
Regorafenib	Stivarga (Bayer)	Multi-TKI (VEGFRs, PDGFRs, FGFRs, TIE2, KIT, RET, RAF)	GIST, CRC, HCC
Pazopanib	Votrient (GlaxoSmithKline)	Multi-TKI (VEGFRs, PDGFRs, FGFR1, c-Kit)	RCC, soft tissue sarcoma
Axitinib	Inlyta (Pfizer)	Multi-TKI (VEGFRs, PDGFRs, c-Kit)	RCC
Vandetanib	Caprelsa (AstraZeneca)	Multi-TKI (VEGFRs, EGFR, RET)	medullary thyroid cancer
Lenvatinib	Lenvima (Eisai)	Multi-TKI (VEGFRs, FGFRs, PDGFRa, RET, c-Kit)	thyroid cancer, RCC
Cabozantinib	Cometriq (Exelixis)/Cabometyx (Exelixis)	Multi-TKI (VEGFRs, cMet, AXL)	medullary thyroid cancer, RCC

*CSFR1, colony stimulating factor 1 receptor; CRC, colorectal cancer; EGFR, epidermal growth factor receptor; FLT3, Fms-like tyrosine kinase 3; GBM, glioblastoma multiforme; GIST, gastrointestinal stromal tumor; HCC, hepatocellular carcinoma; KIT, stem cell factor receptor; MET, hepatocyte growth factor receptor; NSCLC, non-small cell lung cancer; PDGFR, platelet-derived growth factor receptor; RAF, rapidly accelerated fibrosarcoma; RCC, renal cell carcinoma; RET, rearranged during transfection; VEGFR, vascular endothelial growth factor receptor*.

*Anti-angiogenic therapies currently approved by the US Food and Drug Administration (FDA) for the treatment of malignancies (July 2017)*.

*For reference see http://cancer.gov*.

Aflibercept, the “VEGF-trap,” is a fusion protein engineered by joining the second Ig-like domain of VEGFR1 and the third Ig-like domain of VEGFR2 to a human IgG1 Fc-fragment (Holash et al., 2002). This soluble decoy receptor shows one-to-one high-affinity binding to all isoforms of VEGF and PLGF (Figure 1). Clinical randomized phase III trials using aflibercept were performed for several solid cancers (Ciombor et al., 2013) and the addition of this compound to standard therapies lengthened PFS and OS in mCRC patients who progressed on bevacizumab therapy (Van Cutsem et al., 2012a). FDA approved aflibercept in combination with leucovorin, 5-fluorouracil and irinotecan (FOLFIRI) for treating patients after progression with oxaliplatin-containing regimen (Ciombor et al., 2013; Table 1). Furthermore, promising experimental models propose aflibercept as a promising candidate to treat, hepatocarcinoma (HCC) (Torimura et al., 2016), a highly vascular tumor with the development of neoarteries in parallel with tumor growth.

Ramucirumab is a monoclonal antibody that binds the extracellular domain of VEGFR2 and interferes with VEGF binding to its receptor. FDA and EMA (European Medicines Agency) approved this compound either as single agent or in association with paclitaxel in subjects affected by metastatic gastric and gastroesophageal junction cancer after progression on fluoropyrimidine or platinum containing protocols (Fuchs et al., 2014; Wilke et al., 2014; Figure 1). Subsequently, ramucirumab was approved for the second-line treatment of NSCLC with active disease progression or after platinum-based therapy and for the treatment of mCRC in combination with FOLFIRI in patients whose disease was insensitive to bevacizumab, oxaliplatin and fluoropyrimidine (Table 1).

A number of small molecules inhibiting the TK activity of VEGFR, principally (VEGFR2) have been approved as single therapies (Figure 1). Among this class of agents, the pioneer drugs have been sorafenib and sunitinib. Sorafenib is a multikinase inhibitor that targets VEGFR1-3, PDGFRβ, FLT-3, Ret, c-kit, RAF-1, BRAF (Wilhelm et al., 2004). Due to its anti-proliferative, apoptotic, anti-angiogenic and anti-fibrotic effects, sorafenib is a compound with a potent antitumoral activity. Sorafenib is currently the only approved systemic treatment for HCC (Llovet et al., 2008) and several reports have stressed the role of VEGF in the vascularization process of this neoplasia (Miura et al., 1997). Sorafenib has also been approved for the treatment of advanced renal cell carcinoma (RCC) and thyroid cancers (Table 1). The multi-targeted kinase inhibitor sunitinib (VEGFRs, PDGFRs, FLT3, CSFF1R) has been approved for RCC and pancreatic neuroendocrine tumors (Table 1).

Subsequently, other agents were developed with similar targets but are characterized by better toxicity profiles. This second-generation of multi-kinases inhibitors have improved target affinity and less off target effects thus allowing lower concentrations of active drugs to be administered with significant activity. Regorafenib belongs to this second-generation of oral multikinase inhibitors that blocks the activity of several kinases, including those involved in the regulation of tumor angiogenesis (VEGFR1-3 and TIE2), oncogenesis (KIT, RET, RAF1, BRAF and BRAF^V600E^) and the tumor microenvironment (PDGFRβ and FGFR). Moreover, it has been recently shown that regorafenib also exerts anti-metastatic activity because of its capability to inhibit epithelial-mesenchymal transition (Fan et al., 2016). This drug represents a significant improvement over the first-generation of TKI due to its higher specific activity leading to greater pharmacology potency (Wilhelm et al., 2011). Recently, a phase III study showed that regorafenib extended OS and PFS in mCRC patients previously progressed on standard therapies (Grothey et al., 2013). Regorafenib is now approved for the treatment of mCRC and gastrointestinal stromal tumors (Demetri et al., 2013; Table 1).

Among the second-generation multi-kinases class of inhibitors also pazopanib (Gupta and Spiess, 2013), cabozanitinib (Singh et al., 2017), lenvatinib (Fala, 2015), axitinib (Tyler, 2012), and vandetanib (Degrauwe et al., 2012) have been approved as single therapies in specific indications (Table 1).

Recently, based on the result of the phase III LUME-Lung 1 trial (Reck et al., 2014) EMA, but not FDA, approved the use of nintedanib, an oral multi-kinases inhibitor, targeting VEGFR1-3, FRGFR1-3, PDGFRα-β, RET, FLT3, and Src family kinases, combined with docetaxel for the second-line treatment of NSCLC (Lazzari et al., 2017). Moreover, phase II LUME-Meso trial suggested an improvement of PFS of malignant pleural mesothelioma treated with nintedanib in combination with standard treatments (Scagliotti et al., 2016).

### Anti-angiogenic regimens in adjuvant settings

The use of VEGF pathway inhibitors has been started to investigate in phase II and III trials in adjuvant (post-surgical) and neoadjuvant (pre-surgical) settings. Anti-angiogenic agents are used in the adjuvant setting according to the concept that halting angiogenesis after the removal of primary tumor may prevent local relapse micrometastasis spreading tumors. However further clinical and preclinical findings raise doubts on the efficacy of VEGF pathway inhibitors in this setting (Ebos and Kerbel, 2011). Actually, many phase III adjuvant trials with VEGF-targeted therapy failed in CRC, breast cancer, RCC and HCC (de Gramont et al., 2012; Cameron et al., 2013). The reasons of these disappointing results are largely unknown. Probably the different biology of micrometastases from that of established metastatic disease may alter the response to anti-angiogenic agents (Vasudev and Reynolds, 2014).

In neoadjuvant settings, antiangiogenic treatments are used to downsized a tumor, resulting in potentially surgically treatable lesion. Furthermore, it might be used to reduce the risk of local relapse or metastasis. Interestingly the use of bevacizumab (with chemotherapy) in an neoadjuvant setting showed a pathological complete response in breast tumors (Bear et al., 2012; von Minckwitz et al., 2012; Earl et al., 2015; Sikov et al., 2015). Of interest, the efficacy of bevacizumab in promoting vascular normalization in breast tumors correlated with a high baseline microvessel density (MVD), suggesting that basal MVD is a potential biomarker of response to bevacizumab in breast cancer (Tolaney et al., 2015).

### The combination of anti-angiogenic regimens with chemotherapy

As reported above, anti-angiogenic regimens targeting the excess of angiogenic inducers (e.g., bevacizumab or aflibercept) show clinical benefits when associated with cytotoxic therapies (chemotherapy or radiation). Two different observations sustain this rationale. First, this combined strategy can destroy two separate compartments of tumors: cancer cells and endothelial cells (Teicher, 1996). Furthermore, there is a possible synergistic effect of chemotherapy on endothelial compartment by inhibiting endothelial cell cycle. Metronomic chemotherapy is based on this premise and aims at controlling tumor growth by the frequent administration of conventional chemotherapeutic agents at very low doses to target activated endothelial cells in tumors as well as cancer cells, the advantages of which include minimal adverse effects and a rare chance of developing acquired drug resistance (Kerbel, 2015). Second, the vascular normalizing effects of anti-angiogenic regimen modifies the pharmacokinetics parameters of small molecules and favors the delivery of cytotoxic drugs (Zhou et al., 2008; Emblem et al., 2013).

In contrast to anti-angiogenic compounds neutralizing the excess of angiogenic inducers, TKIs do not show any clinical improvement when administered with standard therapies. For instance, attempts to combine anti-angiogenic TKIs with chemotherapy did not improve PFS in mCRC (Carrato et al., 2013) and metastatic breast cancer (Robert et al., 2011b). Indeed, VEGF receptor TKIs exhibit single-agent activity and are effective as monotherapy, while show toxicity in combination with chemotherapy (Jain et al., 2006).

## Mechanisms of resistance to anti-angiogenic regimens

Despite the partial clinical success VEGF-targeted therapies in cancer, some refractory patients do not respond to the treatments (intrinsic resistance) or undergo to acquired resistance after transitory benefits (Bergers and Hanahan, 2008). The extent of refractoriness differs for VEGF blockers and for different cancer types and metastatic settings. Intrinsic and acquired modes of resistance recognize partially overlapping mechanisms, but on the clinical point of view the later represents the most difficult obstacle to achieve better clinical results with anti-angiogenic regimens.

Here we summarize the principal cellular and molecular mechanisms leading to the cancer resistance to anti-angiogenic compounds.

### The vascular features of the tumors

The development of anti-angiogenic strategies started before the genomic revolution signed by the first description of human genome and was largely based on a reductionist perspectives and approaches. VEGF was identified as the master tumor angiogenic inducer and “sprouting angiogenesis” (i.e., the formation of capillaries from pre-existing vessels by endothelial sprouting triggered by angiogenic inducers and followed by formation of endothelial tubes, which undergo maturation by pericyte recruitment and extracellular matrix remodeling) as the almost unique mode to sustain the tumor vascularization (Bussolino et al., 1997). The ability of a cancer clone to trigger an angiogenic response is strictly dependent on its pattern of genomic alterations (Rak et al., 1995; Arbiser, 2004), which evolve along the time of the disease and under the pressure exerted by pharmacological treatments. This situation can be exacerbated by the recent genomic findings revealing evidence of branched evolution, wherein tumors consist of multiple distinct subclones that share a common ancestor but differ in terms of subtle or deep genomic alterations that occur later in the evolution of the cancer (Swanton and Govindan, 2016). Such subclones may be intermixed within one tumor sample or regionally separated within a primary tumor, between primary and metastatic sites, or between metastatic sites (Abbosh et al., 2017; Jamal-Hanjani et al., 2017).

Moreover, communication circuits between cancer and stroma cells result in the production a plethora of angiogenic inducers that can support vascular growth and fitness in the presence blockers of VEGF action. This scenario can precede the use of anti-VEGF therapy and explain the intrinsic resistance or be triggered by VEGF inhibitors resulting in a mode of adaptive resistance (Jayson et al., 2016).

Pre-clinical studies identified numerous candidates that can substitute VEGF in sustaining tumor angiogenesis and include angiopoietins (Ang), ephrins, fibroblast growth factor-1 (FGF1) and−2 (FGF2) (Casanovas et al., 2005), prokineticin-1 (Bv8) (Shojaei et al., 2007b), hepatocyte growth factor (HGF) (Shojaei et al., 2010; Cascone et al., 2017), IL-8 (Huang et al., 2010), platelet-derived growth factor C (PDGFC) (Crawford et al., 2009), VEGFC (Li et al., 2014), and PLGF (Fischer et al., 2007). Most of these studies also show that co-targeting of VEGF and the candidate factor improves therapeutic response. In support to this, clinical evidence show that circulating levels of pro-angiogenic factors, including FGF2, HGF, PLGF, and PDGF can become elevated in patients related to the development of acquired resistance to VEGF blockade (Kopetz et al., 2010).

A more intricate connection exists between the resistance to anti-VEGF therapies and Dll4/Notch axis. In sprouting angiogenesis the expression of Dll4 and Notch are increased by VEGFA and counteract the its proangiogenic effect (Thurston et al., 2007). Interestingly, up-regulation of Dll4 induces resistance to bevacizumab in GBM preclinical models, and is in turn overcame by Notch inhibition by γ-secretase inhibitors (Li et al., 2011). Moreover, it has been also reported that high Dll4 expression is predictive of favorable clinical response to anti-VEGF regimen in ovarian cancer (Hu et al., 2011).

VEGF blockers and more in general all compounds devised to interfere with an angiogenic inducer halt sprouting angiogenesis. However, established evidences indicate that the tumor mass can be vascularized by vessel co-option, a process wherewith tumor cells simply incorporate pre-existing capillaries from surrounding tissue (Holash et al., 1999). Recently, it has reported in metastases blood supply occurs by the non-angiogenic mechanism of vessel co-option (Donnem et al., 2013; Frentzas et al., 2016). The prevalence of vessel co-option in breast cancer and in liver metastasis of CRC (Frentzas et al., 2016) could explain why anti-angiogenic therapies were poorly effective in approaching metastatic breast cancer and showed a moderate efficacy in metastatic CRC.

Pre-clinical evidences support the role of vessel co-option in the onset of resistance to anti-angiogenic regimens in GBM (Rubenstein et al., 2000), HCC (Kuczynski et al., 2016), and in metastasis to lungs (Bridgeman et al., 2017), lymph nodes (Jeong et al., 2015), and liver (Frentzas et al., 2016). Adjuvant trials in thousands of patients with breast cancer and CRC (de Gramont et al., 2012; Cameron et al., 2013) have been negative probably because micrometastases co-opt existing vessels. Therefore, vessel co-option—mediated blood delivery to the growing tumors and metastases can contribute to both adaptive (e.g., in metastatic disease) and intrinsic resistance.

Besides vessel co-option other tumor vascularization mechanisms have been described and are not sustained by sprouting angiogenesis. They include vascular mimicry, in which tumor cells replace endothelial cells to form the capillary wall; tumor vasculogenesis, which is characterized by the recruitment of endothelial precursors from bone marrow and intussusceptive angiogenesis characterized the duplication of a pre-existing vessel by a splitting mechanism. However the clinical relevance of these mechanisms in mediating resistance to anti-angiogenic compounds remain unclear (Lyden et al., 2001; Semela et al., 2007; Kirschmann et al., 2012).

Finally some tumors but in particular pancreatic ductal adenocarcinomas can exhibit primary refractoriness, manifest as a tumor type that is poorly vascularized with a prominent fibrotic reaction and able to survive in adverse and most probably hypoxic conditions (Ryan et al., 2014). A similar circumstance can explain the lack of the effect of anti-angiogenic regimens in the treatment of prostate cancer (Taverna et al., 2013; Jayson et al., 2016).

### Tumor microenvironment

The features of the stroma cells (leucocytes, pericytes, and fibroblasts) in tumors can deeply influence the initial response to angiogenic-regimens as well as the establishment of acquired resistance.

A wide range of myeloid and lymphoid cells can dynamically visit solid tumors. The presence of M2 polarized macrophages or immature Tie2^+^ monocytes can configure a precise circumstance rendering poorly effective the effects of VEGF blockers and specific myeloid-mediated circuits are activated by anti-VEGF therapies and trigger the onset of acquired resistance (Mantovani and Allavena, 2015). Of notice, refractoriness to antiangiogenic therapies, in GBM patients, is associated with higher numbers of CD68^+^ TAMs and CD11b^+^ myeloid cells and the increase of these populations is associated with poor survival (Lu-Emerson et al., 2013).

In particular, a specific circuit eliciting the acquired resistance to anti-VEGF antibodies has been described and involves CD11b^+^/Gr1^+^myeloid cells and T_H_17 lymphocytes, which represent a subset CD4^+^ T cells producing IL-17. It is plausible to envisage that in response to VEGF removal more T_H_17 are recruited and/or start to produce IL-17, which in turn activates the release of G-CSF from stroma cells (Chung et al., 2013). G-CSF is an angiogenic inducer (Bussolino et al., 1989) and is crucial for the mobilization and recruitment of CD11b^+/^Gr1^+^ population to the cancer microenvironment that are capable of promoting VEGF-independent tumorigenesis (Shojaei et al., 2007a). A second circuit entails Ly6C^lo^ monocytes. Anti-VEGF therapy up-regulates CX3CL1 expression, which facilitates CX3CR1-dependent infiltration of Ly6C^lo^ monocytes. These cells attract neutrophils via CXCL5, resulting in the formation of an immunosuppressive microenvironment with a reduction of cytotoxic T lymphocytes (Jung et al., 2017).

Pericytes are mesenchymal cells with contractile properties that patch the capillary outer surface and play a part in vascular physiology. Pericytes are recruited on vessels by PDGFB/PDGFRβ signaling both in physiologic and pathological conditions (Abramsson et al., 2003). In most tumor, vessels are surrounded by few pericytes, but in others a dense pericyte coat with thick basement membrane is present; such vessels are usually less sensitive to VEGF blockers (Bergers et al., 2003). Of interest, several anti-angiogenic TKIs clinical-approved are efficient blockers of both VEGF and PDGF receptors (e.g., sunitinib, sorafenib, pazopanib) and therefore may interfere in pericyte coverage.

Finally, cancer associated fibroblasts (CAFs) or fibrocytic cells recruited from bone marrow are cells that take part to the acquisition of resistance to VEGF-blockers by producing alternative pro-angiogenic substances (Crawford et al., 2009; Mitsuhashi et al., 2015).

Besides the cellular components of the stroma, the features of extracellular matrix can influence the refractoriness to anti-angiogenic therapies. It has been recently noted in human and mouse models of CRC liver metastatization that anti-VEGF therapy results in abnormal deposition of proteoglycans, in particular hyaluronic acid and sulfated glycosaminoglycans (Rahbari et al., 2016). Interestingly, the depletion of hyaluronic acid results in improved tumor perfusion and treatment efficacy in the mouse model of liver mCRC (Rahbari et al., 2016). These findings parallel the observation that anti-angiogenic therapy increases collagen expression, as a consequence of increased hypoxia, in murine models of pancreatic ductal adenocarcinoma (Aguilera et al., 2014) and HCC (Chen et al., 2014).

### Adaption of tumor cells to stressed conditions

As discussed above, a negative consequence of a prolonged treatment with anti-angiogenic regimens is the reduced blood perfusion and metabolites' exchanges, which evolve in hypoxia and acidosis (Jain, 2014). Besides induction of epithelial-to-mesenchymal program that favors an invasive and metastatic tumor cell phenotype hypoxia is thought to select for tumor cells with cancer stem cell properties that might further mediate resistance to cytotoxic agents (Semenza, 2014). In mouse models, hypoxic stress promoted by short-term treatment with anti-VEGF molecules amplified tumor invasiveness and metastatic progression (Ebos et al., 2009; Loges et al., 2009; Pàez-Ribes et al., 2009). The rationale of this paradox is based on the effect on tumor metabolism exerted by the massive vessel pruning and the reduced blood perfusion. The generated hypoxic and acidotic stresses kill a huge amount of cancer cells, but few of them change their features to survive in these hostile conditions by adapting their metabolism, changing the expression of proton pumps, or through autophagy by activating AMP-kinase (Hu et al., 2012; Xu et al., 2013; Fais et al., 2014).

In particular, HIF1-mediated response favors the selection of more aggressive cancer clones (Semenza, 2009) and their metastatic phenotype (Maione et al., 2012) thus explaining the clinical observation that in some solid tumors anti-angiogenic molecules are effective in increasing PFS but they show a negligible effect on OS.

Furthermore, hypoxia favors an immunosuppressive microenvironment by reducing the activity of cytotoxic T cells and antigen-presenting cells and by skewing the polarization of TAMs toward protumorigenic and immunosuppressive M2 phenotype (Mantovani and Allavena, 2015). In HCC, it was demonstrated that increased hypoxia after sorafenib treatment induced Gr1^+^ myeloid-derived suppressor cell recruitment (Chen et al., 2014).

Several pre-clinical studies report that VEGF-targeted therapy can promote increased tumor invasion and metastasis in a hypoxia-independent manner. It was demonstrated that VEGF suppresses HGF-dependent MET phosphorylation and tumor cell migration through the formation of a VEGFR2/MET heterocomplex. This mechanism could explain why VEGF blockade leads to a proinvasive phenotype in preclinical mouse models of GBM and in a subset of GBM patients treated with bevacizumab (Lu et al., 2012).

### Biomarkers and anti-angiogenic therapies

The clinical efficacy of an anti-angiogenic regimen is based on strategies mainly set-up to monitor the tumor cyto-reduction along chemotherapic and radiotherapic treatments. However, the effect of this kind of treatment does not necessarily induce a rapid reduction of tumor mass detectable by imaging approaches or by analyzing the decrease of plasmatic levels of molecules released by the tumor (specific proteins, microRNA, mutated DNAs). Similarly, the present knowledge does not allow predicting which cancer patient can really benefit of an anti-angiogenic treatment.

Huge efforts have been made to evaluate the potential value of circulating angiogenic inducers to address clinical strategies. However high plasmatic levels of VEGF do not predict a response to anti-VEGF/VEGFR2 compounds, and its fluctuation along the treatment is independent from the clinical efficacy (Kopetz et al., 2010). Recent studies have assessed the potential for other biomarkers detectable in plasma. In particular, the pretreatment levels of soluble VEGFR1 inversely correlated with the outcome of either bevacizumab and TKIs because it acts as an endogenous VEGF trap (Meyerhardt et al., 2012; Zhu et al., 2013). Another postulated biomarker is the increased amount of CXCL12, which increased in subjects who escape to anti-angiogenic regimens (Zhu et al., 2009; Batchelor et al., 2010) while low amount of IL-8 at the baseline seems to predict a poor response to bevacizumab treatment in HCC (Boige et al., 2012). Conversely, low pre-treatment levels of Ang-2 were associated with a prolonged PFS in CRC treated with bevacizumab (Goede et al., 2010). Many other works showed an increase of angiogenic molecules along anti-angiogenic regimens and in particular bevacizumab, suggesting that the VEGF removal can trigger the activation of alternative pathways sustaining vascularization, reviewed in Lambrechts et al. (2013).

An emerging diagnostic area still not investigated in anti-angiogenic regimens is represented by circulating exosomes and their cargos (Wang et al., 2016), including microRNA that are promising markers in oncology (Lin and Gregory, 2015).

A second investigative area is the presence in primary tumors of molecules or vascular features, which can predict the response to angiogenesis inhibitors. Generally speaking, many data have been provided such as microvessel density and the expression of pro-angiogenic molecules (VEGFs, VEGFRs, HGF, PDGFs, chemokines, and Ang) but the results are largely contradictory and poorly robust in term of clinical analysis (Lambrechts et al., 2013). In this context one of the more promising result is the correlation between low level of neuropilin-1 expressed in a large cohort of gastric cancers and the prolonged OS after bevacizumab treatment (Van Cutsem et al., 2012b).

Tumors release a plethora of soluble molecules that have a major impact on the biology of bone marrow. Besides modifying the differentiation and the mobilization in particular of myeloid cells, these molecules can promote the mobilization of endothelial precursors. In particular it has been reported that VEGFA or PLGF released by tumor, through a mechanism dependent on metalloproteinase-9 and soluble Kit ligand, increase the number of these cells in bloodstream, while CXCL12 and CXCR4 receptor favor their retention in perivascular site of injured issues (Kopp et al., 2006). The preclinical observation that the number of circulation endothelial precursors was increased by vascular disrupting molecules (Shaked, 2006), many studies focused on the possibility that these cells could be used to monitor or predict the efficacy of anti-angiogenic drugs. Besides the lack of a solid consensus on their phenotype (Ingram et al., 2005) the clinical data on this approach in clinical oncology are conflicting (Bertolini et al., 2006). For instance anti-angiogenic treatment reduces circulating endothelial cells (Dellapasqua et al., 2008), while metronomic therapy shows an opposite effect (Mancuso et al., 2006).

A further promising area is the role exerted by specific single nucleotide polymorphisms (SNPs) of candidate genes to stratify responder and non-responder patients to anti-angiogenic regimen associated with standard therapies. VEGFR1 rs9582036 associated with an improvement of PFS and OS in patients with metastatic pancreatic adenocarcinoma treated with bevacizumab associated with chemotherapy. On the contrary in renal-clear carcinoma VEGFR1 rs7993418 correlated with PFS but not OS in the bevacizumab group (Lambrechts et al., 2012). Another example was reported in metastatic CRC where VEGFA rs833061 and VEGFR1 rs9513070 respectively associated with the objective response rate and the OS in subjects treated with cytotoxic chemotherapy plus bevacizumab (Sohn et al., 2014). Analysis of genetic variants of other angiogenic-related genes in breast cancer using neoadjuvant bevacizumab in combination with chemotherapy compared to chemotherapy alone showed a correlation between specific SNPs in term of pathologic complete response but not in OS (Makhoul et al., 2017).

In recent years, dynamic contrast-enhanced (DCE)-MRI, which enable non-invasive quantification of microvascular structure and function in tumors, has been extensively evaluated in clinical trials as a biomarker for predicting tumor vascular response to anti-angiogenic treatments (Morotti et al., 2017). VEGF blockade is believed to reduce tumor vascular permeability and perfusion. Significant reductions in capillary permeability have been observed in different studies of bevacizumab and TKI in monotherapy or combination with cytotoxic agents (O'Connor et al., 2012). More recently a further exploitation of DCE-MRI termed vessel architectural imaging allowed the vessel caliber estimation and can be considered a powerful biomarker of the vascular normalization induced by anti-angiogenic therapies (Emblem et al., 2013).

## Combination strategies

The partial effect of anti-angiogenic regimens in human cancers and the wide range of mechanisms sustaining intrinsic and acquired resistance represent a driving force for innovative strategies. For example, the anti-angiogenic regimens could improve their efficacy when associated with compounds targeting other major biological processes (e.g., tumor proliferation or apoptosis). In this context, the combination anti-angiogenic molecules with other approaches such as kinase inhibitors, chemotherapy, DNA repair inhibitors, radiotherapy, and immunotherapy have been reported in many experimental and human settings (Jayson et al., 2016).

Furthermore, nanotechnologies approaches could improve the current pharmacokinetic profiles of anti-angiogenic drugs and favor their selective accumulation in tumors and/or induce a shift the microenvironmental equilibria toward tumor-unfavorable conditions (El-Kenawi and El-Remessy, 2013).

### Targeting simultaneously VEGF and other angiogenic mechanisms

Multiple inhibition of concomitant proangiogenic pathways may hamper cancer resistance or extend PFS. A first example deals the simultaneous or sequential blocking of the VEGF and Ang pathways in order to improve efficacy without increasing toxicity (Monk et al., 2014). The tyrosine kinase (TIE2) receptor is activated by its ligand Ang-1, which stabilizes vessels. Ang-2, which antagonizes Ang-1 effects, is highly expressed in cancer, destabilizing vessels and enabling sprouts under a chemotactic gradient of VEGFA. However, the scenario is more intricate because Ang-2 has a partial agonist activity and has a pro-angiogenic effect independent of its cognate receptor TIE2. Increased amount of Ang-2 may be instrumental in eluding the anti-VEGF therapy. Preclinical and clinical studies in GMB reported that Ang-2 levels declined temporarily following inhibition of the VEGF pathway but later rebounded as tumors became resistant to the therapy (Batchelor et al., 2010; Chae et al., 2010). More recently it has been hypothesized that dual inhibition of VEGF and Ang-2 signaling respectively with TKI cediranib and MEDI3617 (an anti-Ang-2-neutralizing antibody) could prolong the temporal window of vascular normalization and thereby enhances the survival benefit of anti-VEGF therapy in two orthotopic murine model of GBM (Peterson et al., 2016). This combinatorial effect is related to an increased amount of recruited M1 polarized TAMs, which have anti-tumor effects. This observation is further supported by the data of another study showing that concurrent blockade of VEGF and Ang-2, using a bispecific Ang-2/VEGF antibody, similarly increased the M1/M2 ratio compared with VEGF-inhibition alone (Kloepper et al., 2016). These results match previous preclinical studies reporting a greater efficacy of combined VEGF and Ang-2 signaling inhibition as compared to single treatment (Brown et al., 2010; Hashizume et al., 2010; Koh et al., 2010; Daly et al., 2013; Kienast et al., 2013).

A second example is the association between VEGF blockade with therapies targeting FGF. Pan inhibitors of the FGF receptor (FGFR1-3), such as AZD4547 and BGJ398, elicited potent anti-tumor activities in preclinical investigations and are currently being evaluated in clinical trials (Chae et al., 2017). In this context, the dual inhibition of VEGFRs and FGFRs using brivanib produced enduring tumors stasis and angiogenic blockade following the failure of VEGF-targeted therapies (Allen et al., 2011).

A third approach exploits the possibility to target VEGF signals and Notch pathway, which is fundamental in regulation tip-stalk endothelial cell dynamics in sprouting angiogenesis (Jakobsson et al., 2009). Down-modulation of the Notch ligand Dll4 in combination with anti-VEGF therapy results in a greater tumor growth inhibition than with each agent alone in ovarian cancer models (Huang et al., 2016).

Fourth, HGF/c-MET pathway is driver and biomarker of VEGFR-inhibitor resistance in NSCLC. Dual VEGFR/c-MET pathway inhibition provide superior therapeutic benefit by delaying the onset of the resistant phenotype (Cascone et al., 2017). The efficacy of combining MET and VEGF inhibitors showed beneficial effect in murine GBM overexpressing MET (Okuda et al., 2017) and in pancreatic neuroendocrine tumors (Sennino et al., 2012).

Simultaneous inhibition of angiogenesis and vessel co-option may represent a further improvement of current therapeutic approaches. It has been recently reported that inhibition of angiogenesis and vessel co-option, by the knockdown of Arp2/3-mediated cancer cell motility, is more effective than targeting angiogenesis alone in a preclinical orthotopic model of advanced CRC liver metastasis (Frentzas et al., 2016).

Finally, tumor angiogenesis may be also affected and regulated by TGFβ family members, that exert a contradictory role in endothelial cells by inhibiting cell migration and proliferation but also acting as a proangiogenic factor and cooperating with VEGF, PDGF, and FGF in autocrine/paracrine signaling (Guerrero and McCarty, 2017). Preclinical studies have shown the anti-angiogenic effect elicited by the TGFβ inhibition in HCC, CRC, and GBM xenografts (Mazzocca et al., 2009; Zhang et al., 2011; Akbari et al., 2014) offering the rationale for the combination of TGFβ inhibitors with VEGF targeting agents (Neuzillet et al., 2015). In particular, are under clinical investigation the efficacy of the combination of galunisertib, a small molecule inhibitor of TGFβRI, with sorafenib and ramucirumab in HCC and PF-03446962, a monoclonal antibody against TGFβ, in combination with regorafenib in CRC.

### Targeting simultaneously VEGF and oncogenic drivers

Different oncogenic hits can perturb the balance between pro- an anti-angiogenic molecules thereby promoting pathological angiogenesis (Arbiser, 2004). For example, MAPK and PI3K-AKT pathways, which are often altered in cancers, are strictly connected with an increased transcription or translation of angiogenic factors. Consequently, specific inhibitors of signaling nodes of these pathways can induce vascular normalization and improve blood perfusion and tumor oxygenation (Qayum et al., 2009).

In particular, RAS activation increases VEGF and IL8 levels and the inhibition of RAS activity by gene silencing suppresses VEGF expression (Mizukami et al., 2005; Matsuo et al., 2009). Moreover, when VEGF expression is inhibited in CRC cells harboring KRAS mutations it has been reported a reduction of *in vivo* tumorigenic potential, highlighting the relevance of VEGF in exploiting the oncogenic potential of mutated KRAS (Okada et al., 1998). The role of KRAS in supporting angiogenesis is confirmed in NSCLC, where VEGF expression correlates with KRAS activating mutations (Konishi et al., 2000). We also described how mutated BRAF affected tumor angiogenesis and proved that targeting BRAF^V600E^ stabilized the tumor vascular bed and abrogated hypoxia in mouse xenografts (Bottos et al., 2012). It has been suggested that EGFR-driven intracellular signaling may control angiogenesis and pharmacological inhibition of EGFR reduces VEGF expression in cancer cells (Ciardiello et al., 2001). It has been reported that a mechanism of acquired resistance to EGFR inhibitors is mediated by the increased secretion of VEGF, suggesting a key role for tumor-induced angiogenesis in the development of anti-EGFR resistance (Ciardiello et al., 2004). In NSCLC preclinical models it was found possible overcome acquired resistance to EGFR inhibitors by adding a VEGF blocker (Naumov et al., 2009). Human epidermal growth factor receptor 2 (HER2) is an oncogene overexpressed in more malignant breast cancer. Trastuzumab, which targets HER2-positive tumors strongly affect vascular shape and function and caused vessel normalization, down-regulating the secretion of VEGF and Ang-1 and in parallel up-regulating the expression of the anti-angiogenic factor thrombospondin 1 (Izumi et al., 2002).

These data suggest that pharmacological inhibition of oncogenes in tumor cells can restore a functional vasculature and potentially blocks the specific angiogenic program activated by individual tumors. Alternative strategy to target tumor angiogenesis could rescue the equilibrium of angiogenic signals by targeting the mutated oncogenes, which play a central role in this process. In order to potentially reduce acquired resistance combined strategy of anti-angiogenic and target therapies are explored in the recent years in pre-clinical and clinical trials.

**Figure 2:**
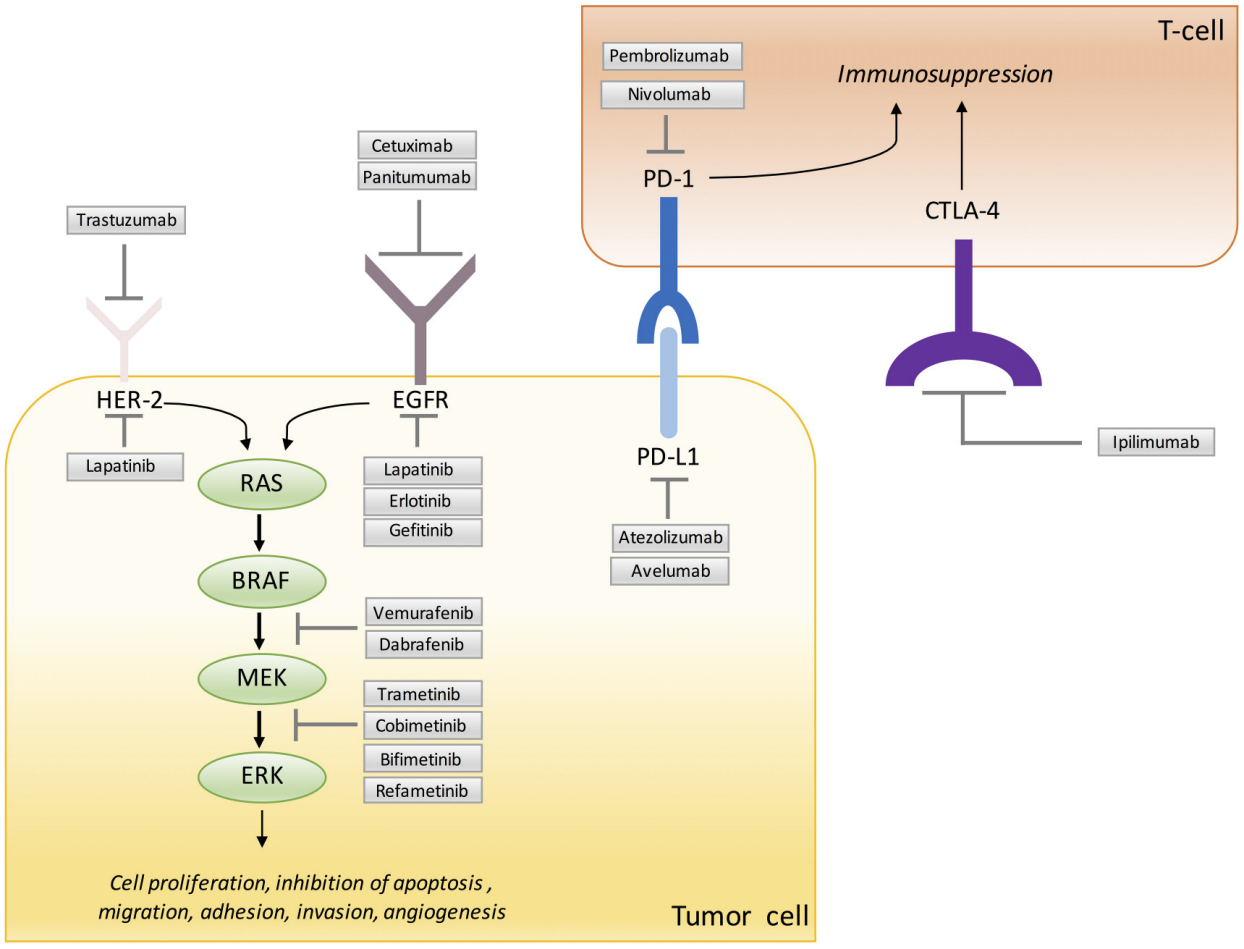
Signaling molecules and immune checkpoint blocked by targeted therapy.

Cetuximab and panitumumab are monoclonal antibodies that block the activation of EGFR and downstream RAS-RAF-MAPK and the PTEN-PIK3CA-AKT pathways (Ciardiello and Tortora, 2008; Figure 2). These two drugs are currently approved for the treatment of mCRC patients with all-*RAS* wild-type tumors. It has been recently reported that combined treatment with cetuximab and regorafenib induced synergistic anti-proliferative and pro-apoptotic effects by blocking MAPK and AKT pathways in orthotopic CRC xenograft models with primary or acquired resistance to anti-EGFR (Napolitano et al., 2015). This beneficial effect can be dependent on the inhibitor activities of regorafenib on different tyrosine kinase receptors involved in angiogenesis and potentially in the mechanism of resistance to cetuximab. The results provide the rationale for the clinical development of this combination. A phase I study was designed to evaluate the antitumor property of this combination among patients with advanced cancer refractory to several lines of therapy (Table 2). This study demonstrated that the combination of regorafenib and cetuximab showed a clinical benefit in all patients. It a plausible that inhibition of one of the molecular targets of regorafenib contributes to overcome resistance to previous anti-VEGF or anti-EGFR therapy (Subbiah et al., 2017). These results sustain the results of a previous work showing the cooperative antitumor activity of cetuximab or erlotinib and sorafenib in a xenograft model of NSCLC (Martinelli et al., 2010). More recently, it has been also shown the prolonged antitumor activity exerted by the combination of erlotinib with bevacizumab in a xenograft model of EGFR-mutated NSCLC (Masuda et al., 2017).

**Table 2:** Selected Clinical Trials of VEGF-targeted therapy in combination with oncogene-targeted therapy (July 2017).

**Anti-angiogenic**	**Target Therapy**	**Phase**	**Indications**	**ClinicalTrials.gov Identifier**
Bevacizumab	Trastuzumab	2	Stage IV metastatic breast cancer	NCT00428922
Bevacizumab	Trastuzumab	3	Metastatic HER2+ breast cancer	NCT00391092
Bevacizumab	Trastuzumab	2	Breast cancer	NCT01321775
Bevacizumab	Trastuzumab	2	Metastatic HER2+ breast cancer	NCT00364611
Bevacizumab	Trastuzumab	2	Metastatic HER2+ breast cancer	NCT00670982
Bevacizumab	Trastuzumab	2	Metastatic HER2+ breast cancer	NCT00392392
Bevacizumab	Trastuzumab	2	Metastatic breast cancer	NCT00405938
Sorafenib	Trametinib	1	HCC	NCT02292173
Sorafenib	Refametinib	2	HCC	NCT01204177
Sorafenib	Refametinib	2	HCC RAS-mutated	NCT01915602
Regorafenib	Refametinib	1	Neoplasm	NCT02168777
Bevacizumab	Erlotinib	3	CRC	NCT00265824
Bevacizumab	Erlotinib	2	NSCLC EGFR-mutated	NCT01562028
Bevacizumab	Erlotinib	2	NSCLC EGFR-mutated	NCT01532089
Regorafenib	Cetuximab	1	Advanced cancers	NCT02095054
Sorafenib	Cetuximab	2	Squamos cell carcinoma of the Head and Neck	NCT00815295
Sorafenib	Cetuximab	2	CRC	NCT00326495
Bevacizumab	Trastuzumab	3	HER2-positive breast cancer	NCT00625898
Pazopanib	Lapatinib	2	HER2-positive breast cancer	NCT00558103.

*CRC, colorectal cancer; EGFR, epiderma growth factor receptor; HCC, hepatocellular carcinoma; HER2, human epidermal growth factor receptor 2; NSCLC, non-small cell lung cancer*.

*For reference see https://clinicaltrials.gov*.

In the clinical setting, several studies are exploring the possibility of combining anti-EGFR drugs such as cetuximab, panitumumab or erlotinib, with different antiangiogenic drugs, including bevacizumab or sorafenib (Table 2). The results in unselected NSCLC or CRC cancer patients have been contradictory. Two large randomized phase III studies have evaluated the efficacy of the addition of cetuximab (CAIRO-2) or panitumumab (PACCE) to an oxaliplatin-containing chemotherapy doublet plus bevacizumab (Hecht et al., 2009; Tol et al., 2009). Both studies showed that the addition of the anti-EGFR antibodies did not improve the therapeutic efficacy. However, the results of randomized phase II study in NSCLC cancer patients selected for the presence of activating EGFR gene mutations demonstrated a clinically relevant increase of PFS by the combined treatment with erlotinib associated with bevacizumab compared erlotinib alone (Seto et al., 2014). This study provided the first evidence that the addition of bevacizumab to erlotinib confers a significant clinical improvement when used as first-line treatment for patients with NSCLC carrying activating EGFR mutations. More recently, the BELIEF trial (NCT01562028) provided further evidences of benefit for the combined use of erlotinib and bevacizumab in patients with EGFR-mutant NSCLC. Of notice, this study was stratified by the presence of the pretreatment of the T790M mutation with EGFR TKI (Rosell et al., 2017). Further, the efficacy and safety of sorafenib and cetuximab association are under evaluation also in patients with head and neck cancer and CRC (Table 2).

It has been demonstrated a positive correlation between elevated HER2 and VEGF levels and the poor outcome of breast cancer (Konecny et al., 2004). Trastuzumab, a monoclonal antibody against HER2, induces normalization and regression of the vasculature in HER2-overexpressing human breast cancer xenografts by lowering the amount of expressed proangiogenic molecules while up-regulating thrombospondin-1, which has anti-angiogenic activity (Izumi et al., 2002; Figure 2). Moreover, in a breast cancer xenograft model, VEGF was found to be elevated in the trastuzumab-resistant group, and sensitivity to trastuzumab was restored upon treatment with bevacizumab (Rugo, 2004). The small molecule inhibitor lapatinib, which inhibits EGFR and HER2, associated with regorafenib showed a greater anti-tumor activity than the compound alone in xenograft models of CRC associated with a relevant reduction of angiogenesis (Zhang et al., 2017). The result of this study has provided the rationale for using HER2 and VEGF inhibitors in clinical practice. Two large phase III trials evaluated the efficacy of bevacizumab and trastuzumab with chemotherapy in HER2^+^ metastatic breast cancer (BETH and AVAREL) (Table 2). A modest improvement was seen in PFS, but the most intriguing finding was a more specific benefit from bevacizumab in the subgroup of patients with high levels of plasmatic VEGFA (Gianni et al., 2013). Moreover, the inhibition of VEGFRs and PDGFRs by pazopanib has been assessed in a phase II trial in combination with lapatinib in HER2-positive breast cancer. In this study the combination of lapatinib and pazopanib was associated with a numerically higher response rate without increase in PFS (Cristofanilli et al., 2013; Table 2).

We have recently demonstrated that targeting the vascular compartment with bevacizumab modulated the response to BRAF^V600E^ inhibition in melanoma and CRC xenograft models. The final result is a synergistic antitumor effect and a delay of the appearance of the acquired resistance to BRAF inhibition. Of interest, we highlighted that this effect is the result of two biological processes: 1) the recruitment of TAMs polarized toward an M1-like phenotype and 2) the stroma remodeling characterized by the reduction of collagen deposition and the number of activated and tumor associated fibroblasts (Comunanza et al., 2017).

In the recent years, there has been great interest in developing clinically effective small-molecule inhibitors of the Ras-Raf-MEK-ERK1/2 pathway (Samatar and Poulikakos, 2014). Recently, (Bridgeman et al., 2016) provide preclinical evidence that combining TKI, such as sunitinib or pazopanib, with a MEK inhibitor (MEKI) is a rationale and efficacious treatment regimen for RCC, showing a more effective suppression of tumor growth and tumor angiogenesis *in vivo*. Furthermore, it has also been reported the enhanced antitumor activity of a new combination regimen containing MEK inhibitor, binimetinib (MEK162), paclitaxel and bevacizumab in platinum-relapsing ovarian patient-derived xenografts (PDX) (Ricci et al., 2017). These results support the ongoing clinical development of MEK inhibitors and VEGF targeted combination therapy (Table 2). In particular, a phase II clinical trial (NCT01204177) investigating refametinib, a potent MEK1/2 inhibitor, in combination with sorafenib as a first-line treatment for subjects with advanced HCC showed that the combination of the two drugs appeared clinically active. Of notice, the majority of patients who responded to this regimen had mutant KRAS tumors (Lim et al., 2014). Further clinical trials are currently being conducted to explore this observation (Table 2).

### Targeting VEGF in combination with immunotherapy

Immune checkpoint inhibition is exploiting in various tumors with promising results. The programmed death protein 1 (PD-1), its ligand the programmed death ligand 1 (PD-L1) and the cytotoxic T-lymphocyte-associated antigen 4 (CTLA-4) are negative regulators of T-cell immune function (Figure 2). Direct stimulation of the immune system with immune check-point inhibitors, such as antibody against PD1-1/PD-L1 and CTLA-4 has been reported in multiple cancers, resulting in several promising clinical trials (Mahoney et al., 2015; Callahan et al., 2016). Despite these exciting results, clinical responses are of limited duration (Sharma and Allison, 2017). A challenging aspect for the development of immune-therapies will be their inclusion in the current therapeutic strategies. Notably, tumor vasculature is an important co-regulator of the immune system and different anti-angiogenesis pathways interact with antitumor immunity through multiple mechanisms (Motz et al., 2014). Of great interest, VEGF was recognized as one of the critical molecule of immune suppression. VEGF reduces adhesion molecules expression on endothelial cells, such as intercellular adhesion molecule-1 (ICAM-1) and vascular adhesion molecule-1 (VCAM-1) impairing leukocyte-endothelial interactions and leukocyte entry in injured tissues. This process results in a dysfunctional tumor vasculature and hinders the immune T effector cell infiltration into the tumors (Ohm, 2003; Motz et al., 2014) and correlates with increased PD-1 expression on CD8 T cells (Voron et al., 2015). In addition to direct effects on T cells, VEGF suppresses dendritic cell differentiation and activity (Gabrilovich et al., 1998) and expands T regulatory cell (Terme et al., 2013) and myeloid-derived suppressor cells (Huang et al., 2007). In patients with CRC bevacizumab improved the antigen-presenting capacity of circulating dendritic cells (Osada et al., 2008), revealing an additional mechanism for bevacizumab on immune functions in the context of checkpoint blockade. Interestingly, it has been recently shown that high serum levels of VEGF were associated with decreased OS in advanced melanoma patients treated with ipilimumab, an anti-CTLA4 antibody (Yuan et al., 2014). In line with this, VEGF was decreased in patients with metastatic melanoma responding to sequential anti-CTLA4 and anti-PD-1 therapy but increased in non-responders (Chen et al., 2016) indicating a mechanism of therapeutic resistance and a potential target to therapy (Ott et al., 2015; Voron et al., 2015).

Besides to the effects on tumor vasculature, VEGF blockade may have a positive impact on the immune mechanisms leading to an anti-tumor response and preclinical studies support the possibility to exploit anti-angiogenesis inhibitors in association with molecule regulating innate and adaptive immunity. It has been reported in preclinical models of melanoma that blockade of the VEGF/VEGFR2 pathway increased the anti-tumor activity of adoptively transferred T-cells (Shrimali et al., 2010) and the combination of blocking VEGFR2 by the specific monoclonal antibody DC101 with a cancer vaccination showed a great anti-tumor effect by favoring CD8^+^ T cell recruitment and reducing the number of regulatory T cells, which have tumor immune-suppressive function (Huang et al., 2012).

The positive effect on immune response obtained by halting VEGF pathway can be further increased by combining the block of Ang-2. A bispecific antibody, which bind both VEGFA and Ang-2 showed a better effect as compared to the single block, in many pre-clinical models and synergized with PD-1 blockade. Mechanistically, the antagonistic effect on these two angiogenic molecules favors the vascular normalization with a more efficient recruitment of CD8^+^ T, which is concomitantly characterized by the up-regulation of PD-L1 on perivascular T cells (Schmittnaegel et al., 2017).

Further, the addition of anti-PD1 antibody to the CXCR4 inhibitor AMD3100 and sorafenib augments the antitumor immune responses mediated by CD8^+^ T cells in an orthotopic murine models of HCC. The triple association showed a significant activity both on primary tumors and on the lung metastatic spreading (Chen et al., 2015).

More recently, a preclinical study provided evidences that anti-PD-1 or anti PD-L1 therapy sensitized and prolonged the efficacy of antiangiogenic therapy, and conversely, antiangiogenic therapy improved anti-PD-L1 treatment by supporting vascular changes, such as vessel normalization and high endothelial venules formation, that facilitate enhanced cytotoxic T cell infiltration and subsequent tumor cell destruction (Allen et al., 2017).

Based on these preclinical and translational data supporting synergy between angiogenesis inhibitors and checkpoint blockers, multiple trials of combinatorial therapies are under way and some have produced encouraging results. For example a phase I trial data of combination of bevacizumab and ipilimumab in patients with advanced melanoma showed disease control and increased CD8 T-cell tumor infiltration, resulting in durable patient response of more than 6 months (Hodi et al., 2014; Ott et al., 2015).

Other clinical trials are evaluating the combination between anti-angiogenic regimens and antibody targeting PD1 (nivolumab, pembrolizumab) and PDL1 (MPDL-3280A) (Table 3).

**Table 3:** Selected Clinical Trials of VEGF-targeted therapy in combination with immune checkpoint inhibitors (July 2017).

**Anti-angiogenic**	**Immunotherapy**	**Phase**	**Indications**	**ClinicalTrials.gov Identifier**
Bevacizumab	Ipilimumab	2	Melanoma	NCT01950390
Bevacizumab	Ipilimumab	1	Melanoma	NCT00790010
Bevacizumab	Atezolizumab	2	CRC	NCT02982694
Bevacizumab	Atezolizumab	2	Melanoma brain metastases	NCT03175432
Bevacizumab	Atezolizumab	2	RCC	NCT02724878
Bevacizumab	Atezolizumab	3	RCC	NCT02420821
Bevacizumab	Nivolumab	2	Ovarian, Fallopian Tube Or Peritoneal Cancer	NCT02873962
Bevacizumab	Nivolumab	3	Glioblastoma	NCT02017717
Bevacizumab	Nivolumab	1	NSCLC	NCT01454102
Bevacizumab	Nivolumab	1	RCC	NCT02210117
Bevacizumab	Pembrolizumab	2	RCC	NCT02348008
Bevacizumab	Pembrolizumab	1/2	NSCLC	NCT02039674
Bevacizumab	Pembrolizumab	2	Glioblastoma	NCT02337491
Bevacizumab	Pembrolizumab	2	Melanoma/NSCLC brain metastases	NCT02681549
Aflibercept	Pembrolizumab	1	Solid tumors	NCT02298959
Sunitinib	Nivolumab	1	RCC	NCT01472081
Axitinib	Pembrolizumab	3	RCC	NCT02853331
Axitinib	Avelumab	3	RCC	NCT02684006
Cabozantinib	Nivolumab	3	RCC	NCT03141177

*CRC, colorectal cancer; NSCLC, non-small cell lung cancer; RCC, renal cell carcinoma*.

*For reference see https://clinicaltrials.gov*.

## Conclusion

Preclinical findings show that single-drug antiangiogenic therapy delayed tumor growth but it was unable to determine tumor regression (Jayson et al., 2016) and in general, clinical efficacy of anti-angiogenic agents is lower than that observed in preclinical cancer models (Ebos and Kerbel, 2011) with significant adverse effects. The mechanisms that restrain the therapeutic efficacy of anti-angiogenic drugs in cancer are still poor comprehended. Moreover, an essential issue in the smart development of these compound is the identification of predictive biomarkers to find responder and non-responder patients. However biomarkers that are predictive of response to anti-angiogenic therapy in patients remain elusive (Jain et al., 2009; Vasudev and Reynolds, 2014; Jayson et al., 2016) and the patients' stratification on the basis of the drivers mutations and on feature of transcriptomic landscape including both gene coding and non-coding RNAs could really ameliorate the selection of responder patients.

Furthermore, biomarkers analysis and identification could represent the rationale for novel and combinatorial approaches, which could improve the clinical outcome exerted by angiogenesis inhibition. In particular oncogenes and immune response play a central role in the regulation of tumor angiogenesis and for this reason represent two attractive targets to develop combinatorial strategies. Many preclinical studies encourage the clinical exploitation of this approach.

## Author contributions

VC contributed to the research, figure design, and writing of manuscript. FB contributed to the research, editing, and overall design of manuscript.

### Conflict of interest statement

The authors declare that the research was conducted in the absence of any commercial or financial relationships that could be construed as a potential conflict of interest. The handling Editor declared a past co-authorship with one of the author FB.

## References

[B1] Abbosh C. Birkbak N. J. Wilson G. A. Jamal-Hanjani M. Constantin T. Salari R.. (2017). Phylogenetic ctDNA analysis depicts early-stage lung cancer evolution. Nature 545, 446–451. 10.1038/nature22364 28445469 PMC5812436

[B2] Abramsson A. Lindblom P. Betsholtz C. (2003). Endothelial and nonendothelial sources of PDGF-B regulate pericyte recruitment and influence vascular pattern formation in tumors. J. Clin. Invest. 112, 1142–1151. 10.1172/JCI200318549 14561699 PMC213487

[B3] Aguilera K. Y. Rivera L. B. Hur H. Carbon J. G. Toombs J. E. Goldstein C. D.. (2014). Collagen signaling enhances tumor progression after anti-VEGF therapy in a murine model of pancreatic ductal adenocarcinoma. Cancer Res. 74, 1032–1044. 10.1158/0008-5472.CAN-13-2800 24346431 PMC3944405

[B4] Akbari A. Amanpour S. Muhammadnejad S. Ghahremani M. H. Ghaffari S. H. Dehpour A. R.. (2014). Evaluation of antitumor activity of a TGF-beta receptor I inhibitor (SD-208) on human colon adenocarcinoma. DARU J. Pharm. Sci. 22:47. 10.1186/2008-2231-22-47 24902843 PMC4077684

[B5] Allen E. Jabouille A. Rivera L. B. Lodewijckx I. Missiaen R. Steri V.. (2017). Combined antiangiogenic and anti–PD-L1 therapy stimulates tumor immunity through HEV formation. Sci. Transl. Med. 9:eaak9679. 10.1126/scitranslmed.aak9679 28404866 PMC5554432

[B6] Allen E. Walters I. B. Hanahan D. (2011). Brivanib, a dual FGF/VEGF inhibitor, is active both first and second line against mouse pancreatic neuroendocrine tumors developing adaptive/evasive resistance to VEGF inhibition. Clin. Cancer Res. 17, 5299–5310. 10.1158/1078-0432.CCR-10-2847 21622725 PMC3156934

[B7] Arbiser J. L. (2004). Molecular regulation of angiogenesis and tumorigenesis by signal transduction pathways: evidence of predictable and reproducible patterns of synergy in diverse neoplasms. Semin. Cancer Biol. 14, 81–91. 10.1016/j.semcancer.2003.09.013 15018892

[B8] Batchelor T. T. Duda D. G. di Tomaso E. Ancukiewicz M. Plotkin S. R. Gerstner E.. (2010). Phase II study of cediranib, an oral pan-vascular endothelial growth factor receptor tyrosine kinase inhibitor, in patients with recurrent glioblastoma. J. Clin. Oncol. 28, 2817–2823. 10.1200/JCO.2009.26.3988 20458050 PMC2903316

[B9] Batchelor T. T. Gerstner E. R. Emblem K. E. Duda D. G. Kalpathy-Cramer J. Snuderl M.. (2013). Improved tumor oxygenation and survival in glioblastoma patients who show increased blood perfusion after cediranib and chemoradiation. Proc. Natl. Acad. Sci. U.S.A. 110, 19059–19064. 10.1073/pnas.1318022110 24190997 PMC3839699

[B10] Bear H. D. Tang G. Rastogi P. Geyer C. E. Robidoux A. Atkins J. N.. (2012). Bevacizumab added to neoadjuvant chemotherapy for breast cancer. N. Engl. J. Med. 366, 310–320. 10.1056/NEJMoa1111097 22276821 PMC3401076

[B11] Bergers G. Hanahan D. (2008). Modes of resistance to anti-angiogenic therapy. Nat. Rev. Cancer 8, 592–603. 10.1038/nrc2442 18650835 PMC2874834

[B12] Bergers G. Song S. Meyer-Morse N. Bergsland E. Hanahan D. (2003). Benefits of targeting both pericytes and endothelial cells in the tumor vasculature with kinase inhibitors. J. Clin. Invest. 111, 1287–1295. 10.1172/JCI200317929 12727920 PMC154450

[B13] Bertolini F. Shaked Y. Mancuso P. Kerbel R. S. (2006). The multifaceted circulating endothelial cell in cancer: towards marker and target identification. Nat. Rev. Cancer 6, 835–845. 10.1038/nrc1971 17036040

[B14] Boige V. Malka D. Bourredjem A. Dromain C. Baey C. Jacques N.. (2012). Efficacy, Safety, and Biomarkers of Single-Agent Bevacizumab Therapy in Patients with Advanced Hepatocellular Carcinoma. Oncology 17, 1063–1072. 10.1634/theoncologist.2011-0465 22707516 PMC3425524

[B15] Bottos A. Martini M. Di Nicolantonio F. Comunanza V. Maione F. Minassi A.. (2012). Targeting oncogenic serine/threonine-protein kinase BRAF in cancer cells inhibits angiogenesis and abrogates hypoxia. Proc. Natl. Acad. Sci. U.S.A. 109, E353–E359. 10.1073/pnas.1105026109 22203991 PMC3277561

[B16] Bottsford-Miller J. N. Coleman R. L. Sood A. K. (2012). Resistance and escape from antiangiogenesis therapy: clinical implications and future strategies. J. Clin. Oncol. 30, 4026–4034. 10.1200/JCO.2012.41.9242 23008289 PMC3488272

[B17] Bridgeman V. L. Vermeulen P. B. Foo S. Bilecz A. Daley F. Kostaras E.. (2017). Vessel co-option is common in human lung metastases and mediates resistance to anti-angiogenic therapy in preclinical lung metastasis models. J. Pathol. 241, 362–374. 10.1002/path.4845 27859259 PMC5248628

[B18] Bridgeman V. L. Wan E. Foo S. Nathan M. R. Welti J. C. Frentzas S.. (2016). Preclinical evidence that trametinib enhances the response to antiangiogenic tyrosine kinase inhibitors in renal cell carcinoma. Mol. Cancer Ther. 15, 172–183. 10.1158/1535-7163.MCT-15-0170 26487278

[B19] Brown J. L. Cao Z. A. Pinzon-Ortiz M. Kendrew J. Reimer C. Wen S.. (2010). A Human Monoclonal Anti-ANG2 antibody leads to broad antitumor activity in combination with VEGF inhibitors and chemotherapy agents in preclinical models. Mol. Cancer Ther. 9, 145–156. 10.1158/1535-7163.MCT-09-0554 20053776

[B20] Brufsky A. M. Hurvitz S. Perez E. Swamy R. Valero V. O'Neill V.. (2011). RIBBON-2: a randomized, double-blind, placebo-controlled, phase III trial evaluating the efficacy and safety of bevacizumab in combination with chemotherapy for second-line treatment of human epidermal growth factor receptor. J. Clin. Oncol. 29, 4286–4293. 10.1200/JCO.2010.34.1255 21990397

[B21] Bussolino F. Mantovani A. Persico G. (1997). Molecular mechanisms of blood vessel formation. Trends Biochem. Sci. 22, 251–256. 10.1016/S0968-0004(97)01074-8 9255066

[B22] Bussolino F. Wang J. M. Defilippi P. Turrini F. Sanavio F. Edgell C. J.. (1989). Granulocyte- and granulocyte- macrophage-colony stimulating factors induce human endothelial cells to migrate and proliferate. Nature 337, 471–473. 10.1038/337471a0 2464767

[B23] Callahan M. K. Postow M. A. Wolchok J. D. (2016). Targeting T cell co-receptors for cancer therapy. Immunity 44, 1069–1078. 10.1016/j.immuni.2016.04.023 27192570

[B24] Cameron D. Brown J. Dent R. Jackisch C. Mackey J. Pivot X.. (2013). Adjuvant bevacizumab-containing therapy in triple-negative breast cancer (BEATRICE): primary results of a randomised, phase 3 trial. Lancet Oncol. 14, 933–942. 10.1016/S1470-2045(13)70335-8 23932548

[B25] Carmeliet P. Jain R. K. (2000). Angiogenesis in cancer and other diseases. Nature 407, 249–257. 10.1038/35025220 11001068

[B26] Carmeliet P. Jain R. K. (2011). Principles and mechanisms of vessel normalization for cancer and other angiogenic diseases. Nat. Rev. Drug Discov. 10, 417–427. 10.1038/nrd3455 21629292

[B27] Carrato A. Swieboda-Sadlej A. Staszewska-Skurczynska M. Lim R. Roman L. Shparyk Y. (2013). Fluorouracil, leucovorin, and irinotecan plus either sunitinib or placebo in metastatic colorectal cancer: a randomized, phase III trial. J. Clin. Oncol. 31, 1341–1347. 10.1200/JCO.2012.45.1930 23358972

[B28] Casanovas O. Hicklin D. J. Bergers G. Hanahan D. (2005). Drug resistance by evasion of antiangiogenic targeting of VEGF signaling in late-stage pancreatic islet tumors. Cancer Cell 8, 299–309. 10.1016/j.ccr.2005.09.005 16226705

[B29] Cascone T. Xu L. Lin H. Y. Liu W. Tran H. T. Liu Y.. (2017). The HGF/c-MET pathway is a driver and biomarker of VEGFR-inhibitor resistance and vascular remodeling in non-small cell lung cancer. Clin. Cancer Res. Clincanres. 23, 5489–5501. 10.1158/1078-0432.CCR-16-3216 28559461 PMC5600821

[B30] Chae S. S. Kamoun W. S. Farrar C. T. Kirkpatrick N. D. Niemeyer E. de Graaf A. M.. (2010). Angiopoietin-2 interferes with anti-VEGFR2-induced vessel normalization and survival benefit in mice bearing gliomas. Clin. Cancer Res. 16, 3618–3627. 10.1158/1078-0432.CCR-09-3073 20501615 PMC2905497

[B31] Chae Y. K. Ranganath K. Hammerman P. S. Vaklavas C. Mohindra N. Kalya A.. (2017). Inhibition of the fibroblast growth factor receptor (FGFR) pathway: the current landscape and barriers to clinical application. Oncotarget 8, 16052–16074. 10.18632/oncotarget.14109 28030802 PMC5362545

[B32] Chen P. L. Roh W. Reuben A. Cooper Z. A. Spencer C. N. Prieto P. A.. (2016). Analysis of immune signatures in longitudinal tumor samples yields insight into biomarkers of response and mechanisms of resistance to immune checkpoint blockade. Cancer Discov. 6, 827–837. 10.1158/2159-8290.CD-15-1545 27301722 PMC5082984

[B33] Chen Y. Huang Y. Reiberger T. Duyverman A. M. Huang P. Samuel R.. (2014). Differential effects of sorafenib on liver versus tumor fibrosis mediated by stromal-derived factor 1 alpha/C-X-C receptor type 4 axis and myeloid differentiation antigen-positive myeloid cell infiltration in mice. Hepatology 59, 1435–1447. 10.1002/hep.26790 24242874 PMC3966948

[B34] Chen Y. Ramjiawan R. R. Reiberger T. Ng M. R. Hato T. Huang Y.. (2015). CXCR4 inhibition in tumor microenvironment facilitates anti-programmed death receptor-1 immunotherapy in sorafenib-treated hepatocellular carcinoma in mice. Hepatology 61, 1591–1602. 10.1002/hep.27665 25529917 PMC4406806

[B35] Chung A. S. Wu X. Zhuang G. Ngu H. Kasman I. Zhang J.. (2013). An interleukin-17–mediated paracrine network promotes tumor resistance to anti-angiogenic therapy. Nat. Med. 19, 1114–1123. 10.1038/nm.3291 23913124

[B36] Ciardiello F. Bianco R. Caputo R. Caputo R. Damiano V. Troiani T.. (2004). Antitumor activity of ZD6474, a vascular endothelial growth factor receptor tyrosine kinase inhibitor, in human cancer cells with acquired resistance to antiepidermal growth factor receptor therapy. Clin. Cancer Res. 10, 784–793. 10.1158/1078-0432.CCR-1100-03 14760102

[B37] Ciardiello F. Caputo R. Bianco R. Damiano V. Fontanini G. Cuccato S.. (2001). Inhibition of growth factor production and angiogenesis in human cancer cells by ZD1839 (Iressa), a selective epidermal growth factor receptor tyrosine kinase inhibitor. Clin. Cancer Res. 7, 1459–1465. 11350918

[B38] Ciardiello F. Tortora G. (2008). EGFR antagonists in cancer treatment. N. Engl. J. Med. 358, 1160–1174. 10.1056/NEJMra0707704 18337605

[B39] Ciombor K. K. Berlin J. Chan E. (2013). Aflibercept. Clin. Cancer Res. 19, 1920–1925. 10.1158/1078-0432.CCR-12-2911 23444216 PMC3710732

[B40] Comunanza V. Corà D. Orso F. Consonni F. M. Middonti E. Di Nicolantonio F. (2017). VEGF blockade enhances the antitumor effect of BRAF^V 600E^ inhibition. EMBO Mol. Med. 9, 219–237. 10.15252/emmm.201505774 27974353 PMC5286370

[B41] Crawford Y. Kasman I. Yu L. Zhong C. Wu X. Modrusan Z.. (2009). PDGF-C mediates the angiogenic and tumorigenic properties of fibroblasts associated with tumors refractory to anti-VEGF treatment. Cancer Cell 15, 21–34. 10.1016/j.ccr.2008.12.004 19111878

[B42] Cristofanilli M. Johnston S. R. Manikhas A. Gomez H. L. Gladkov O. Shao Z.. (2013). A randomized phase II study of lapatinib + pazopanib versus lapatinib in patients with HER2+ inflammatory breast cancer. Breast Cancer Res. Treat. 137, 471–482. 10.1007/s10549-012-2369-x 23239151 PMC3539065

[B43] Cunningham D. Lang I. Marcuello E. Lorusso V. Ocvirk J. Shin D. B.. (2013). Bevacizumab plus capecitabine versus capecitabine alone in elderly patients with previously untreated metastatic colorectal cancer (AVEX): An open-label, randomised phase 3 trial. Lancet Oncol. 14, 1077–1085. 10.1016/S1470-2045(13)70154-2 24028813

[B44] Daly C. Eichten A. Castanaro C. Pasnikowski E. Adler A. Lalani A. S.. (2013). Angiopoietin-2 functions as a Tie2 agonist in tumor models, where it limits the effects of VEGF inhibition. Cancer Res. 73, 108–118. 10.1158/0008-5472.CAN-12-2064 23149917

[B45] de Gramont A. Van Cutsem E. Schmoll H. J. Tabernero J. Clarke S. Moore M. J.. (2012). Bevacizumab plus oxaliplatin-based chemotherapy as adjuvant treatment for colon cancer (AVANT): a phase 3 randomised controlled trial. Lancet Oncol. 13, 1225–1233. 10.1016/S1470-2045(12)70509-0 23168362

[B46] Degrauwe N. Sosa J. A. Roman S. Deshpande H. A. (2012). Vandetanib for the treatment of metastatic medullary Thyroid Cancer. Clin. Med. Insights. Oncol. 6, 243–252. 10.4137/CMO.S7999 22723734 PMC3379848

[B47] Dellapasqua S. Bertolini F. Bagnardi V. Campagnoli E. Scarano E. Torrisi R.. (2008). Metronomic Cyclophosphamide and Capecitabine Combined with Bevacizumab in Advanced Breast Cancer. J. Clin. Oncol. 26, 4899–4905. 10.1200/JCO.2008.17.4789 18794539

[B48] Demetri G. D. Reichardt P. Kang Y. K. Blay J. Y. Rutkowski P. Gelderblom H.. (2013). Efficacy and safety of regorafenib for advanced gastrointestinal stromal tumours after failure of imatinib and sunitinib (GRID): an international, multicentre, randomised, placebo-controlled, phase 3 trial. Lancet 381, 295–302. 10.1016/S0140-6736(12)61857-1 23177515 PMC3819942

[B49] Donnem T. Hu J. Ferguson M. Adighibe O. Snell C. Harris A. L.. (2013). Vessel co-option in primary human tumors and metastases: an obstacle to effective anti-angiogenic treatment? Cancer Med. 2, 427–436. 10.1002/cam4.105 24156015 PMC3799277

[B50] Earl H. M. Hiller L. Dunn J. A. Blenkinsop C. Grybowicz L. Vallier A. L.. (2015). Efficacy of neoadjuvant bevacizumab added to docetaxel followed by fluorouracil, epirubicin, and cyclophosphamide, for women with HER2-negative early breast cancer (ARTemis): an open-label, randomised, phase 3 trial. Lancet Oncol. 16, 656–666. 10.1016/S1470-2045(15)70137-3 25975632

[B51] Ebos J. M. Kerbel R. S. (2011). Antiangiogenic therapy: impact on invasion, disease progression, and metastasis. Nat. Rev. Clin. Oncol. 8, 210–221. 10.1038/nrclinonc.2011.21 21364524 PMC4540336

[B52] Ebos J. M. L. Lee C. R. Cruz-Munoz W. Bjarnason G. A. Christensen J. G. Kerbel R. S. (2009). Accelerated metastasis after short-term treatment with a potent inhibitor of tumor angiogenesis. Cancer Cell 15, 232–239. 10.1016/j.ccr.2009.01.021 19249681 PMC4540346

[B53] El-Kenawi A. E. El-Remessy A. B. (2013). Angiogenesis inhibitors in cancer therapy: mechanistic perspective on classification and treatment rationales. Br. J. Pharmacol. 170, 712–729. 10.1111/bph.12344 23962094 PMC3799588

[B54] Emblem K. E. Mouridsen K. Bjornerud A. Farrar C. T. Jennings D. Borra R. J.. (2013). Vessel architectural imaging identifies cancer patient responders to anti-angiogenic therapy. Nat. Med. 19, 1178–1183. 10.1038/nm.3289 23955713 PMC3769525

[B55] Fais S. Venturi G. Gatenby B. (2014). Microenvironmental acidosis in carcinogenesis and metastases: new strategies in prevention and therapy. Cancer Metastasis Rev. 33, 1095–1108. 10.1007/s10555-014-9531-3 25376898 PMC4244550

[B56] Fala L. (2015). Lenvima (Lenvatinib), a multireceptor tyrosine kinase inhibitor, approved by the FDA for the treatment of patients with differentiated Thyroid Cancer. Am. Heal. Drug Benefits 8, 176–179. 26629286 PMC4665059

[B57] Fan L. C. Teng H. W. Shiau C. W. Tai W. T. Hung M. H. Yang S. H.. (2016). Regorafenib (Stivarga) pharmacologically targets epithelial- mesenchymal transition in colorectal cancer. Oncotarget 7, 64136–64147. 10.18632/oncotarget.11636 27580057 PMC5325431

[B58] Ferrara N. (2002). VEGF and the quest for tumour angiogenesis factors. Nat. Rev. Cancer 2, 795–803. 10.1038/nrc909 12360282

[B59] Ferrara N. Kerbel R. S. (2005). Angiogenesis as a therapeutic target. Nature 438, 967–974. 10.1038/nature04483 16355214

[B60] Ferrara N. Hillan K. J. Gerber H.-P. Novotny W. (2004). Discovery and development of bevacizumab, an anti-VEGF antibody for treating cancer. Nat. Rev. Drug Discov. 3, 391–400. 10.1038/nrd1381 15136787

[B61] Fischer C. Jonckx B. Mazzone M. Zacchigna S. Loges S. Pattarini L.. (2007). Anti-PlGF inhibits growth of VEGF(R)-inhibitor-resistant tumors without affecting healthy vessels. Cell 131, 463–475. 10.1016/j.cell.2007.08.038 17981115

[B62] Folkman J. (1971). Tumor angiogenesis: therapeutic implications. N. Engl. J. Med. 285, 1182–1186. 10.1056/NEJM197111182852108 4938153

[B63] Folkman J. (2006). Angiogenesis. Annu. Rev. Med. 57, 1–18. 10.1146/annurev.med.57.121304.131306 16409133

[B64] Frentzas S. Simoneau E. Bridgeman V. L. Vermeulen P. B. Foo S. Kostaras E.. (2016). Vessel co-option mediates resistance to anti-angiogenic therapy in liver metastases. Nat. Med. 22, 1294–1302. 10.1038/nm.4197 27748747 PMC5104270

[B65] Friedman H. S. Prados M. D. Wen P. Y. Mikkelsen T. Schiff D. Abrey L. E.. (2009). Bevacizumab alone and in combination with irinotecan in recurrent glioblastoma. J. Clin. Oncol. 27, 4733–4740. 10.1200/JCO.2008.19.8721 19720927

[B66] Fuchs C. S. Tomasek J. Yong C. J. Dumitru F. Passalacqua R. Goswami C.. (2014). Ramucirumab monotherapy for previously treated advanced gastric or gastro-oesophageal junction adenocarcinoma (REGARD): an international, randomised, multicentre, placebo-controlled, phase 3 trial. Lancet 383, 31–39. 10.1016/S0140-6736(13)61719-5 24094768

[B67] Gabrilovich D. Ishida T. Oyama T. Ran S. Kravtsov V. Nadaf S.. (1998). Vascular endothelial growth factor inhibits the development of dendritic cells and dramatically affects the differentiation of multiple hematopoietic lineages *in vivo*. Blood 92, 4150–4166. 9834220

[B68] Gianni L. Romieu G. H. Lichinitser M. Serrano S. V. Mansutti M. Pivot X.. (2013). AVEREL: a randomized phase III Trial evaluating bevacizumab in combination with docetaxel and trastuzumab as first-line therapy for HER2-positive locally recurrent/metastatic breast cancer. J. Clin. Oncol. 31, 1719–1725. 10.1200/JCO.2012.44.7912 23569311

[B69] Giantonio B. J. Catalano P. J. Meropol N. J. O'Dwyer P. J. Mitchell E. P. Alberts S. R.. (2007). Bevacizumab in combination with oxaliplatin, fluorouracil, and leucovorin (FOLFOX4) for previously treated metastatic colorectal cancer: results from the Eastern Cooperative Oncology Group Study E3200. J. Clin. Oncol. 25, 1539–1544. 10.1200/JCO.2006.09.6305 17442997

[B70] Goede V. Coutelle O. Neuneier J. Reinacher-Schick A. Schnell R. Koslowsky T. C.. (2010). Identification of serum angiopoietin-2 as a biomarker for clinical outcome of colorectal cancer patients treated with bevacizumab-containing therapy. Br. J. Cancer 103, 1407–1414. 10.1038/sj.bjc.6605925 20924372 PMC2990609

[B71] Goel S. Duda D. G. Xu L. Munn L. L. Boucher Y. Fukumura D.. (2011). Normalization of the vasculature for treatment of cancer and other diseases. Physiol. Rev. 91, 1071–1121. 10.1152/physrev.00038.2010 21742796 PMC3258432

[B72] Grothey A. Van Cutsem E. Sobrero A. Siena S. Falcone A. Ychou M.. (2013). Regorafenib monotherapy for previously treated metastatic colorectal cancer (CORRECT): an international, multicentre, randomised, placebo-controlled, phase 3 trial. Lancet 381, 303–312. 10.1016/S0140-6736(12)61900-X 23177514

[B73] Guerrero P. A. McCarty J. H. (2017). TGF-β activation and signaling in angiogenesis, in Physiologic and Pathologic Angiogenesis - Signaling Mechanisms and Targeted Therapy, eds Simionescu D. Simionescu A. (Rijeka: InTech). 10.5772/66405

[B74] Gupta S. Spiess P. E. (2013). The prospects of pazopanib in advanced renal cell carcinoma. Ther. Adv. Urol. 5, 223–232. 10.1177/1756287213495099 24082917 PMC3763778

[B75] Hanahan D. Folkman J. (1996). Patterns and emerging mechanisms of the angiogenic switch during tumorigenesis. Cell 86, 353–364. 10.1016/S0092-8674(00)80108-7 8756718

[B76] Hashizume H. Falcón B. L. Kuroda T. Baluk P. Coxon A. Yu D.. (2010). Complementary actions of inhibitors of angiopoietin-2 and VEGF on tumor angiogenesis and growth. Cancer Res. 70, 2213–2223. 10.1158/0008-5472.CAN-09-1977 20197469 PMC2840050

[B77] Hecht J. R. Mitchell E. Chidiac T. Scroggin C. Hagenstad C. Spigel D.. (2009). A randomized phase IIIB trial of chemotherapy, bevacizumab, and panitumumab compared with chemotherapy and bevacizumab alone for metastatic colorectal cancer. J. Clin. Oncol. 27, 672–680. 10.1200/JCO.2008.19.8135 19114685

[B78] Hodi F. S. Lawrence D. Lezcano C. Wu X. Zhou J. Sasada T.. (2014). Bevacizumab plus ipilimumab in patients with metastatic melanoma. Cancer Immunol. Res. 2, 632–642. 10.1158/2326-6066.CIR-14-0053 24838938 PMC4306338

[B79] Holash J. Davis S. Papadopoulos N. Croll S. D. Ho L. Russell M.. (2002). VEGF-Trap: a VEGF blocker with potent antitumor effects. Proc. Natl. Acad. Sci. U.S.A. 99, 11393–11398. 10.1073/pnas.172398299 12177445 PMC123267

[B80] Holash J. Maisonpierre P. C. Compton D. Boland P. Alexander C. R. Zagzag D.. (1999). Vessel cooption, regression, and growth in tumors mediated by angiopoietins and VEGF. Science 284, 1994–1998. 10.1126/science.284.5422.1994 10373119

[B81] Hu W. Lu C. Dong H. H. Huang J. Shen D. Y. Stone R. L.. (2011). Biological roles of the delta family notch ligand Dll4 in tumor and endothelial cells in ovarian cancer. Cancer Res. 71, 6030–6039. 10.1158/0008-5472.CAN-10-2719 21795478 PMC3174342

[B82] Hu Y. L. DeLay M. Jahangiri A. Molinaro A. M. Rose S. D. Carbonell W. S.. (2012). Hypoxia-induced autophagy promotes tumor cell survival and adaptation to antiangiogenic treatment in glioblastoma. Cancer Res. 72, 1773–1783. 10.1158/0008-5472.CAN-11-3831 22447568 PMC3319869

[B83] Huang D. Ding Y. Zhou M. Rini B. I. Petillo D. Qian C. N.. (2010). Interleukin-8 mediates resistance to antiangiogenic agent sunitinib in renal cell carcinoma. Cancer Res. 70, 1063–1071. 10.1158/0008-5472.CAN-09-3965 20103651 PMC3719378

[B84] Huang J. Hu W. Hu L. Previs R. A. Dalton H. J. Yang X. Y.. (2016). Dll4 Inhibition plus Aflibercept Markedly Reduces Ovarian Tumor Growth. Mol. Cancer Ther. 15, 1344–1352. 10.1158/1535-7163.MCT-15-0144 27009216 PMC4893925

[B85] Huang Y. Chen X. Dikov M. M. Novitskiy S. V. Mosse C. A. Yang L.. (2007). Distinct roles of VEGFR-1 and VEGFR-2 in the aberrant hematopoiesis associated with elevated levels of VEGF. Blood 110, 624–631. 10.1182/blood-2007-01-065714 17376891 PMC1924481

[B86] Huang Y. Goel S. Duda D. G. Fukumura D. Jain R. K. (2013). Vascular normalization as an emerging strategy to enhance cancer immunotherapy. Cancer Res. 73, 2943–2948. 10.1158/0008-5472.CAN-12-4354 23440426 PMC3655127

[B87] Huang Y. Yuan J. Righi E. Kamoun W. S. Ancukiewicz M. Nezivar J.. (2012). Vascular normalizing doses of antiangiogenic treatment reprogram the immunosuppressive tumor microenvironment and enhance immunotherapy. Proc. Natl. Acad. Sci. U.S.A. 109, 17561–17566. 10.1073/pnas.1215397109 23045683 PMC3491458

[B88] Hurwitz H. Fehrenbacher L. Novotny W. Cartwright T. Hainsworth J. Heim W.. (2004). Bevacizumab plus irinotecan, fluorouracil, and leucovorin for metastatic colorectal cancer. N. Engl. J. Med. 350, 2335–2342. 10.1056/NEJMoa032691 15175435

[B89] Ingram D. A. Caplice N. M. Yoder M. C. (2005). Unresolved questions, changing definitions, and novel paradigms for defining endothelial progenitor cells. Blood 106:1525 LP-1531. 10.1182/blood-2005-04-1509 15905185

[B90] Izumi Y. Xu L. di Tomaso E. Fukumura D. Jain R. K. (2002). Tumour biology: herceptin acts as an anti-angiogenic cocktail. Nature 416, 279–280. 10.1038/416279b 11907566

[B91] Jain R. K. (2005). Normalization of tumor vasculature: an emerging concept in antiangiogenic therapy. Science 307, 58–62. 10.1126/science.1104819 15637262

[B92] Jain R. K. (2014). Antiangiogenesis strategies revisited: from starving tumors to alleviating hypoxia. Cancer Cell 26, 605–622. 10.1016/j.ccell.2014.10.006 25517747 PMC4269830

[B93] Jain R. K. Duda D. G. Clark J. W. Loeffler J. S. (2006). Lessons from phase III clinical trials on anti-VEGF therapy for cancer. Nat. Clin. Pract. Oncol. 3, 24–40. 10.1038/ncponc0403 16407877

[B94] Jain R. K. Duda D. G. Willett C. G. Sahani D. V. Zhu A. X. Loeffler J. S.. (2009). Biomarkers of response and resistance to antiangiogenic therapy. Nat. Rev. Clin. Oncol. 6, 327–338. 10.1038/nrclinonc.2009.63 19483739 PMC3057433

[B95] Jakobsson L. Bentley K. Gerhardt H. (2009). VEGFRs and Notch: a dynamic collaboration in vascular patterning. Biochem. Soc. Trans. 37, 1233–1236. 10.1042/BST0371233 19909253

[B96] Jamal-Hanjani M. Wilson G. A. McGranahan N. Birkbak N. J. Watkins T. B. K. Veeriah S.. (2017). Tracking the evolution of non–small-cell lung cancer. N. Engl. J. Med. 376, 2109–2121. 10.1056/NEJMoa1616288 28445112

[B97] Jayson G. C. Kerbel R. Ellis L. M. Harris A. L. (2016). Antiangiogenic therapy in oncology: current status and future directions. Lancet 388, 518–529. 10.1016/S0140-6736(15)01088-0 26853587

[B98] Jeong H. S. Jones D. Liao S. Wattson D. A. Cui C. H. Duda D. G.. (2015). Investigation of the lack of angiogenesis in the formation of lymph node metastases. J. Natl. Cancer Inst. 107:djv155. 10.1093/jnci/djv155 26063793 PMC4651102

[B99] Jung K. Heishi T. Khan O. F. Kowalski P. S. Incio J. Rahbari N. N.. (2017). Ly6Clo monocytes drive immunosuppression and confer resistance to anti-VEGFR2 cancer therapy. J. Clin. Invest. 127, 3039–3051. 10.1172/JCI93182 28691930 PMC5531423

[B100] Kamoun W. S. Ley C. D. Farrar C. T. Duyverman A. M. Lahdenranta J. Lacorre D. A.. (2009). Edema control by cediranib, a vascular endothelial growth factor receptor–targeted kinase inhibitor, prolongs survival despite persistent brain tumor growth in mice. J. Clin. Oncol. 27, 2542–2552. 10.1200/JCO.2008.19.9356 19332720 PMC2739611

[B101] Kerbel R. (2008). Tumor angiogenesis. N. Engl. J. Med. 358, 2039–2049. 10.1056/NEJMra0706596 18463380 PMC4542009

[B102] Kerbel R. S. (2015). A decade of experience in developing preclinical models of advanced- or early-stage spontaneous metastasis to study antiangiogenic drugs, metronomic chemotherapy, and the tumor microenvironment. Cancer J. 21, 274–283. 10.1097/PPO.0000000000000134 26222079

[B103] Kienast Y. Klein C. Scheuer W. Raemsch R. Lorenzon E. Bernicke D.. (2013). Ang-2-VEGF-A crossmab, a novel bispecific human IgG1 antibody Blocking VEGF-A and Ang-2 functions simultaneously, mediates potent antitumor, antiangiogenic, and antimetastatic efficacy. Clin. Cancer Res. 19, 6730–6740. 10.1158/1078-0432.CCR-13-0081 24097868

[B104] Kirschmann D. A. Seftor E. A. Hardy K. M. Seftor R. E. Hendrix M. J. (2012). Molecular pathways: vasculogenic mimicry in tumor cells: diagnostic and therapeutic implications. Clin. Cancer Res. 18, 2726–2732. 10.1158/1078-0432.CCR-11-3237 22474319 PMC3354024

[B105] Kloepper J. Riedemann L. Amoozgar Z. Seano G. Susek K. Yu V.. (2016). Ang-2/VEGF bispecific antibody reprograms macrophages and resident microglia to anti-tumor phenotype and prolongs glioblastoma survival. Proc. Natl. Acad. Sci. U.S.A. 113, 4476–4481. 10.1073/pnas.1525360113 27044098 PMC4843473

[B106] Koh Y. J. Kim H. Z. Hwang S. I. Lee J. E. Oh N. Jung K.. (2010). Double antiangiogenic protein, DAAP, targeting VEGF-A and angiopoietins in tumor angiogenesis, metastasis, and vascular leakage. Cancer Cell 18, 171–184. 10.1016/j.ccr.2010.07.001 20708158

[B107] Konecny G. E. Meng Y. G. Untch M. Wang H.-J. Bauerfeind I. Epstein M.. (2004). Association between HER-2/*neu* and vascular endothelial growth factor expression predicts clinical outcome in primary breast cancer patients. Clin. Cancer Res. 10, 1706–1716. 10.1158/1078-0432.CCR-0951-3 15014023

[B108] Konishi T. Huang C. L. Adachi M. Taki T. Inufusa H. Kodama K.. (2000). The K-ras gene regulates vascular endothelial growth factor gene expression in non-small cell lung cancers. Int. J.Oncol. 16, 501–511. 10.3892/ijo.16.3.501 10675482

[B109] Kopetz S. Hoff P. M. Morris J. S. Wolff R. A. Eng C. Glover K. Y.. (2010). Phase II trial of infusional fluorouracil, irinotecan, and bevacizumab for metastatic colorectal cancer: efficacy and circulating angiogenic biomarkers associated with therapeutic resistance. J. Clin. Oncol. 28, 453–459. 10.1200/JCO.2009.24.8252 20008624 PMC2815707

[B110] Kopp H. G. Ramos C. A. Rafii S. (2006). Contribution of endothelial progenitors and proangiogenic hematopoietic cells to vascularization of tumor and ischemic tissue. Curr. Opin. Hematol. 13, 175–181. 10.1097/01.moh.0000219664.26528.da 16567962 PMC2945883

[B111] Kuczynski E. A. Yin M. Bar-Zion A. Lee C. R. Butz H. Man S. (2016). Co-option of liver vessels and not sprouting angiogenesis drives acquired sorafenib resistance in hepatocellular carcinoma. J. Natl. Cancer Inst. 108:djw030 10.1093/jnci/djw030 PMC5017954 27059374

[B112] Kwilas A. R. Donahue R. N. Tsang K. Y. Hodge J. W. (2015). Immune consequences of tyrosine kinase inhibitors that synergize with cancer immunotherapy. Cancer Cell Microenviron. 2, 1–11. 10.14800/ccm.677 26005708 PMC4440700

[B113] Lambrechts D. Claes B. Delmar P. Reumers J. Mazzone M. Yesilyurt B. T.. (2012). VEGF pathway genetic variants as biomarkers of treatment outcome with bevacizumab: an analysis of data from the AViTA and AVOREN randomised trials. Lancet Oncol. 13, 724–733. 10.1016/S1470-2045(12)70231-0 22608783

[B114] Lambrechts D. Lenz H.-J. de Haas S. Carmeliet P. Scherer S. J. (2013). Markers of response for the antiangiogenic agent bevacizumab. J. Clin. Oncol. 31, 1219–1230. 10.1200/JCO.2012.46.2762 23401453

[B115] Lazzari C. Karachaliou N. Gregorc V. Bulotta A. Gonzalez-Cao M. Verlicchi A.. (2017). Second-line therapy of squamous non-small cell lung cancer: an evolving landscape. Expert Rev. Respir. Med. 11, 469–479. 10.1080/17476348.2017.1326822 28467720

[B116] Li D. Xie K. Ding G. Li J. Chen K. Li H.. (2014). Tumor resistance to anti-VEGF therapy through up-regulation of VEGF-C expression. Cancer Lett. 346, 45–52. 10.1016/j.canlet.2013.12.004 24333721

[B117] Li J. L. Sainson R. C. A. Oon C. E. Turley H. Leek R. Sheldon H.. (2011). DLL4-Notch signaling mediates tumor resistance to anti-VEGF therapy *in vivo*. Cancer Res. 71, 6073–6083. 10.1158/0008-5472.CAN-11-1704 21803743

[B118] Lim H. Y. Heo J. Choi H. J. Lin C.-Y. Yoon J.-H. Hsu C.. (2014). A phase II study of the efficacy and safety of the combination therapy of the MEK inhibitor refametinib (BAY 86-9766) Plus Sorafenib for Asian Patients with Unresectable Hepatocellular Carcinoma. Clin. Cancer Res. 20, 5976–5985. 10.1158/1078-0432.CCR-13-3445 25294897

[B119] Lin S. Gregory R. I. (2015). MicroRNA biogenesis pathways in cancer. Nat Rev Cancer 15, 321–333. 10.1038/nrc3932 25998712 PMC4859809

[B120] Llovet J. M. Ricci S. Mazzaferro V. Hilgard P. Gane E. Blanc J. F.. (2008). Sorafenib for advanced hepatocellular carcinoma. N. Engl. J. Med. 359, 378–390. 10.1056/NEJMoa0708857 18650514

[B121] Loges S. Mazzone M. Hohensinner P. Carmeliet P. (2009). Silencing or fueling metastasis with VEGF inhibitors: antiangiogenesis revisited. Cancer Cell 15, 167–170. 10.1016/j.ccr.2009.02.007 19249675

[B122] Lu K. V. Chang J. P. Parachoniak C. A. Pandika M. M. Aghi M. K. Meyronet D.. (2012). VEGF inhibits tumor cell invasion and mesenchymal transition through a MET/VEGFR2 complex. Cancer Cell 22, 21–35. 10.1016/j.ccr.2012.05.037 22789536 PMC4068350

[B123] Lu-Emerson C. Snuderl M. Kirkpatrick N. D. Goveia J. Davidson C. Huang Y.. (2013). Increase in tumor-associated macrophages after antiangiogenic therapy is associated with poor survival among patients with recurrent glioblastoma. Neuro. Oncol. 15, 1079–1087. 10.1093/neuonc/not082 23828240 PMC3714160

[B124] Lyden D. Hattori K. Dias S. Costa C. Blaikie P. Butros L.. (2001). Impaired recruitment of bone-marrow-derived endothelial and hematopoietic precursor cells blocks tumor angiogenesis and growth. Nat. Med. 7, 1194–1201. 10.1038/nm1101-1194 11689883

[B125] Mahoney K. M. Rennert P. D. Freeman G. J. (2015). Combination cancer immunotherapy and new immunomodulatory targets. Nat. Rev. Drug Discov. 14, 561–584. 10.1038/nrd4591 26228759

[B126] Maione F. Capano S. Regano D. Zentilin L. Giacca M. Casanovas O.. (2012). Semaphorin 3A overcomes cancer hypoxia and metastatic dissemination induced by antiangiogenic treatment in mice. J. Clin. Invest. 122, 1832–1848. 10.1172/JCI58976 22484816 PMC3336974

[B127] Makhoul I. Todorova V. K. Siegel E. R. Erickson S. W. Dhakal I. Raj V. R.. (2017). Germline Genetic Variants in TEK, ANGPT1, ANGPT2, MMP9, FGF2 and VEGFA are associated with pathologic complete response to Bevacizumab in Breast Cancer Patients. PLoS ONE 12:e0168550. 10.1371/journal.pone.0168550 28045923 PMC5207665

[B128] Mancuso P. Colleoni M. Calleri A. Orlando L. Maisonneuve P. Pruneri G.. (2006). Circulating endothelial-cell kinetics and viability predict survival in breast cancer patients receiving metronomic chemotherapy. Blood 108, 452–459. 10.1182/blood-2005-11-4570 16543470 PMC1895485

[B129] Mantovani A. Allavena P. (2015). The interaction of anticancer therapies with tumor-associated macrophages. J. Exp. Med. 212, 435–445. 10.1084/jem.20150295 25753580 PMC4387285

[B130] Martinelli E. Troiani T. Morgillo F. Rodolico G. Vitagliano D. Morelli M. P.. (2010). Synergistic antitumor activity of sorafenib in combination with epidermal growth factor receptor inhibitors in colorectal and lung cancer cells. Clin. Cancer Res. 16, 4990–5001. 10.1158/1078-0432.CCR-10-0923 20810384

[B131] Masuda C. Yanagisawa M. Yorozu K. Kurasawa M. Furugaki K. Ishikura N.. (2017). Bevacizumab counteracts VEGF-dependent resistance to erlotinib in an EGFR-mutated NSCLC xenograft model. Int. J. Oncol. 51, 425–434. 10.3892/ijo.2017.4036 28627678 PMC5504975

[B132] Matsuo Y. Campbell P. M. Brekken R. A. Sung B. Ouellette M. M. Fleming J. B.. (2009). K-Ras promotes angiogenesis mediated by immortalized human pancreatic epithelial cells through mitogen-activated protein kinase signaling pathways. Mol. Cancer Res. 7, 799–808. 10.1158/1541-7786.MCR-08-0577 19509115 PMC4267726

[B133] Mazzocca A. Fransvea E. Lavezzari G. Antonaci S. Giannelli G. (2009). Inhibition of transforming growth factor β receptor I kinase blocks hepatocellular carcinoma growth through neo-angiogenesis regulation. Hepatology 50, 1140–1151. 10.1002/hep.23118 19711426

[B134] Meyerhardt J. A. Ancukiewicz M. Abrams T. A. Schrag D. Enzinger P. C. Chan J. A.. (2012). Phase I Study of Cetuximab, Irinotecan, and Vandetanib (ZD6474) as therapy for patients with previously treated metastastic colorectal cancer. PLoS ONE 7:e38231. 10.1371/journal.pone.0038231 22701615 PMC3373492

[B135] Miles D. W. Chan A. Dirix L. Y. Cortés J. Pivot X. Tomczak P.. (2010). Phase III study of bevacizumab plus docetaxel compared with placebo plus docetaxel for the first-line treatment of human epidermal growth factor receptor 2-negative metastatic breast cancer. J. Clin. Oncol. 28, 3239–3247. 10.1200/JCO.2008.21.6457 20498403

[B136] Miller K. Wang M. Gralow J. Dickler M. Cobleigh M. Perez E. A.. (2007). Paclitaxel plus bevacizumab versus paclitaxel alone for metastatic breast cancer. N. Engl. J. Med. 357, 2666–2676. 10.1056/NEJMoa072113 18160686

[B137] Mitsuhashi A. Goto H. Saijo A. Trung V. T. Aono Y. Ogino H.. (2015). Fibrocyte-like cells mediate acquired resistance to anti-angiogenic therapy with bevacizumab. Nat. Commun. 6:8792. 10.1038/ncomms9792 26635184 PMC4686833

[B138] Miura H. Miyazaki T. Kuroda M. Oka T. Machinami R. Kodama T.. (1997). Increased expression of vascular endothelial growth factor in human hepatocellular carcinoma. J. Hepatol. 27, 854–861. 10.1016/S0168-8278(97)80323-6 9382973

[B139] Mizukami Y. Jo W.-S. Duerr E.-M. Gala M. Li J. Zhang X.. (2005). Induction of interleukin-8 preserves the angiogenic response in HIF-1α-deficient colon cancer cells. Nat. Med. 11, 992–997. 10.1038/nm1294 16127434

[B140] Monk B. J. Poveda A. Vergote I. Raspagliesi F. Fujiwara K. Bae D. S.. (2014). Anti-angiopoietin therapy with trebananib for recurrent ovarian cancer (TRINOVA-1): a randomised, multicentre, double-blind, placebo-controlled phase 3 trial. Lancet Oncol. 15, 799–808. 10.1016/S1470-2045(14)70244-X 24950985

[B141] Morotti M. Dass P. H. Harris A. L. Lord S. (2017). Pharmacodynamic and pharmacokinetic markers for anti-angiogenic cancer therapy: implications for dosing and selection of patients. Eur. J. Drug Metab. Pharmacokinet. [Epub ahead of print]. 10.1007/s13318-017-0442-x 29019020

[B142] Motz G. T. Santoro S. P. Wang L.-P. Garrabrant T. Lastra R. R. Hagemann I. S.. (2014). Tumor endothelium FasL establishes a selective immune barrier promoting tolerance in tumors. Nat. Med. 20, 607–615. 10.1038/nm.3541 24793239 PMC4060245

[B143] Nagy J. A. Feng D. Vasile E. Wong W. H. Shih S.-C. Dvorak A. M.. (2006). Permeability properties of tumor surrogate blood vessels induced by VEGF-A. Lab. Investig. 86, 767–780. 10.1038/labinvest.3700436 16732297

[B144] Napolitano S. Martini G. Rinaldi B. Martinelli E. Donniacuo M. Berrino L.. (2015). Primary and acquired resistance of colorectal cancer to anti-EGFR monoclonal antibody can be overcome by combined treatment of regorafenib with cetuximab. Clin. Cancer Res. 21, 2975–2983. 10.1158/1078-0432.CCR-15-0020 25838391

[B145] Naumov G. N. Nilsson M. B. Cascone T. Briggs A. Straume O. Akslen L. A.. (2009). Combined vascular endothelial growth factor receptor and Epidermal Growth Factor Receptor (EGFR) blockade inhibits tumor growth in xenograft models of EGFR inhibitor resistance. Clin. Cancer Res. 15, 3484–3494. 10.1158/1078-0432.CCR-08-2904 19447865 PMC2893040

[B146] Neuzillet C. Tijeras-Raballand A. Cohen R. Cros J. Faivre S. Raymond E.. (2015). Targeting the TGFβ pathway for cancer therapy. Pharmacol. Ther. 147, 22–31. 10.1016/j.pharmthera.2014.11.001 25444759

[B147] O'Connor J. P. Jackson A. Parker G. J. Roberts C. Jayson G. C. (2012). Dynamic contrast-enhanced MRI in clinical trials of antivascular therapies. Nat. Rev. Clin. Oncol. 9, 167–177. 10.1038/nrclinonc.2012.2 22330689

[B148] Ohm J. E. (2003). VEGF inhibits T-cell development and may contribute to tumor-induced immune suppression. Blood 101, 4878–4886. 10.1182/blood-2002-07-1956 12586633

[B149] Okada F. Rak J. W. Croix B. S. Lieubeau B. Kaya M. Roncari L. (1998). Impact of oncogenes in tumor angiogenesis: mutant K-ras up-regulation of vascular endothelial growth factor/vascular permeability factor is necessary, but not sufficient for tumorigenicity of human colorectal carcinoma cells. Proc. Natl. Acad. Sci. U.S.A. 95, 3609–3614. 10.1073/pnas.95.7.3609 9520413 PMC19883

[B150] Okuda T. Tasaki T. Nakata S. Yamashita K. Yoshioka H. Izumoto S.. (2017). Efficacy of Combination Therapy with MET and VEGF Inhibitors for MET-overexpressing Glioblastoma. Anticancer Res. 37, 3871–3876. 28668888 10.21873/anticanres.11767

[B151] Osada T. Chong G. Tansik R. Hong T. Spector N. Kumar R.. (2008). The effect of anti-VEGF therapy on immature myeloid cell and dendritic cells in cancer patients. Cancer Immunol. Immunother. 57, 1115–1124. 10.1007/s00262-007-0441-x 18193223 PMC4110970

[B152] Ott P. A. Hodi F. S. Buchbinder E. I. (2015). Inhibition of immune checkpoints and vascular endothelial growth factor as combination therapy for metastatic melanoma: an overview of rationale, preclinical evidence, and initial clinical data. Front. Oncol. 5:202. 10.3389/fonc.2015.00202 26442214 PMC4585112

[B153] Pàez-Ribes M. Allen E. Hudock J. Takeda T. Okuyama H. Viñals F. (2009). Antiangiogenic therapy elicits malignant progression of tumors to increased local invasion and distant metastasis. Cancer Cell 15, 220–231. 10.1016/j.ccr.2009.01.027 19249680 PMC2874829

[B154] Peterson T. E. Kirkpatrick N. D. Huang Y. Farrar C. T. Marijt K. A. Kloepper J.. (2016). Dual inhibition of Ang-2 and VEGF receptors normalizes tumor vasculature and prolongs survival in glioblastoma by altering macrophages. Proc. Natl. Acad. Sci. U.S.A. 113, 4470–4475. 10.1073/pnas.1525349113 27044097 PMC4843449

[B155] Qayum N. Muschel R. J. Im J. H. Balathasan L. Koch C. J. Patel S.. (2009). Tumor vascular changes mediated by inhibition of oncogenic signaling. Cancer Res. 69, 6347–6354. 10.1158/0008-5472.CAN-09-0657 19622766 PMC2825046

[B156] Rahbari N. N. Kedrin D. Incio J. Liu H. Ho W. W. Nia H. T.. (2016). Anti-VEGF therapy induces ECM remodeling and mechanical barriers to therapy in colorectal cancer liver metastases. Sci. Transl. Med. 8:360ra135. 10.1126/scitranslmed.aaf5219 27733559 PMC5457741

[B157] Rak J. Filmus J. Finkenzeller G. Grugel S. Marmé D. Kerbel R. S. (1995). Oncogenes as inducers of tumor angiogenesis. Cancer Metastasis. Rev. 14, 263–277. 10.1007/BF00690598 8821090

[B158] Reck M. Kaiser R. Mellemgaard A. Douillard J.-Y. Orlov S. Krzakowski M.. (2014). Docetaxel plus nintedanib versus docetaxel plus placebo in patients with previously treated non-small-cell lung cancer (LUME-Lung 1): a phase 3, double-blind, randomised controlled trial. Lancet Oncol. 15, 143–155. 10.1016/S1470-2045(13)70586-2 24411639

[B159] Ricci F. Guffanti F. Damia G. Broggini M. (2017). Combination of paclitaxel, bevacizumab and MEK162 in second line treatment in platinum-relapsing patient derived ovarian cancer xenografts. Mol. Cancer 16:97. 10.1186/s12943-017-0662-3 28558767 PMC5450309

[B160] Robert N. J. Diéras V. Glaspy J. Brufsky A. M. Bondarenko I. Lipatov O. N. (2011a). RIBBON-1: randomized, double-blind, placebo-controlled, phase III trial of chemotherapy with or without bevacizumab for first-line treatment of human epidermal growth factor receptor 2-negative, locally recurrent or metastatic breast cancer. J. Clin. Oncol. 29, 1252–1260. 10.1200/JCO.2010.28.0982 21383283

[B161] Robert N. J. Saleh M. N. Paul D. Generali D. Gressot L. Copur M. S.. (2011b). Sunitinib plus paclitaxel versus bevacizumab plus paclitaxel for first-line treatment of patients with advanced breast cancer: a phase III, randomized, open-label trial. Clin. Breast Cancer 11, 82–92. 10.1016/j.clbc.2011.03.005 21569994 PMC4617186

[B162] Rosell R. Dafni U. Felip E. Curioni-Fontecedro A. Gautschi O. Peters S.. (2017). Erlotinib and bevacizumab in patients with advanced non-small-cell lung cancer and activating EGFR mutations (BELIEF): an international, multicentre, single-arm, phase 2 trial. Lancet Respir. Med. 5, 435–444. 10.1016/S2213-2600(17)30129-7 28408243

[B163] Rubenstein J. L. Kim J. Ozawa T. Zhang M. Westphal M. Deen D. F.. (2000). Anti-VEGF antibody treatment of glioblastoma prolongs survival but results in increased vascular cooption. Neoplasia (New York, NY) 2, 306–314. 10.1038/sj.neo.7900102 11005565 PMC1550290

[B164] Rugo H. S. (2004). Bevacizumab in the Treatment of Breast Cancer: rationale and current data. Oncologist 9, 43–49. 10.1634/theoncologist.9-suppl_1-43 15178815

[B165] Ryan D. P. Hong T. S. Bardeesy N. (2014). Pancreatic adenocarcinoma. N. Engl. J. Med. 35, 353–354. 10.1056/NEJMra1404198

[B166] Samatar A. A. Poulikakos P. I. (2014). Targeting RAS–ERK signalling in cancer: promises and challenges. Nat. Rev. Drug Discov. 13, 928–942. 10.1038/nrd4281 25435214

[B167] Scagliotti G. V. Gaafar R. Nowak A. Vogelzang N. J. Von Wangenheim U. Morsli N.. (2016). P2.01: LUME-MeSO: Phase II/III Study of Nintedanib + Pemetrexed/Cisplatin in Patients With Malignant Pleural Mesothelioma. J. Thorac. Oncol. 11:S216. 10.1016/j.jtho.2016.08.07527676538

[B168] Schmittnaegel M. Rigamonti N. Kadioglu E. Cassará A. Wyser Rmili C. Kiialainen A.. (2017). Dual angiopoietin-2 and VEGFA inhibition elicits antitumor immunity that is enhanced by PD-1 checkpoint blockade. Sci. Transl. Med. 9:eaak9670. 10.1126/scitranslmed.aak9670 28404865

[B169] Semela D. Piguet A. C. Kolev M. Schmitter K. Hlushchuk R. Djonov V.. (2007). Vascular remodeling and antitumoral effects of mTOR inhibition in a rat model of hepatocellular carcinoma. J. Hepatol. 46, 840–848. 10.1016/j.jhep.2006.11.021 17321636

[B170] Semenza G. L. (2009). Defining the role of hypoxia-inducible factor 1 in cancer biology and therapeutics. Oncogene 29, 625–634. 10.1038/onc.2009.441 19946328 PMC2969168

[B171] Semenza G. L. (2014). Oxygen sensing, hypoxia-inducible factors, and disease pathophysiology. Annu. Rev. Pathol. 9, 47–71. 10.1146/annurev-pathol-012513-104720 23937437

[B172] Sennino B. Ishiguro-Oonuma T. Wei Y. Naylor R. M. Williamson C. W. Bhagwandin V.. (2012). Suppression of tumor invasion and metastasis by concurrent inhibition of c-Met and VEGF signaling in pancreatic neuroendocrine tumors. Cancer Discov. 2, 270–287. 10.1158/2159-8290.CD-11-0240 22585997 PMC3354652

[B173] Seto T. Kato T. Nishio M. Goto K. Atagi S. Hosomi Y.. (2014). Erlotinib alone or with bevacizumab as first-line therapy in patients with advanced non-squamous non-small-cell lung cancer harbouring EGFR mutations (JO25567): an open-label, randomised, multicentre, phase 2 study. Lancet Oncol. 15, 1236–1244. 10.1016/S1470-2045(14)70381-X 25175099

[B174] Shaked Y. (2006). Therapy-induced acute recruitment of circulating endothelial progenitor cells to tumors. Science 313, 1785–1787. 10.1126/science.1127592 16990548

[B175] Sharma P. Allison J. P. (2017). Immune checkpoint targeting in cancer therapy: toward combination strategies with curative potential. Cell 161, 205–214. 10.1016/j.cell.2015.03.030 25860605 PMC5905674

[B176] Shojaei F. Lee J. H. Simmons B. H. Wong A. Esparza C. O. Plumlee P. A.. (2010). HGF/c-Met acts as an alternative angiogenic pathway in sunitinib-resistant tumors. Cancer Res. 70, 10090–10100. 10.1158/0008-5472.CAN-10-0489 20952508

[B177] Shojaei F. Wu X. Malik A. K. Zhong C. Baldwin M. E. Schanz S.. (2007a). Tumor refractoriness to anti-VEGF treatment is mediated by CD11b^+^Gr1^+^ myeloid cells. Nat. Biotechnol. 25, 911–920. 10.1038/nbt1323 17664940

[B178] Shojaei F. Wu X. Zhong C. Yu L. Liang X.-H. Yao J.. (2007b). Bv8 regulates myeloid-cell-dependent tumour angiogenesis. Nature 450, 825–831. 10.1038/nature06348 18064003

[B179] Shrimali R. K. Yu Z. Theoret M. R. Chinnasamy D. Restifo N. P. Rosenberg S. A. (2010). Antiangiogenic agents can increase lymphocyte infiltration into tumor and enhance the effectiveness of adoptive immunotherapy of cancer. Cancer Res. 70, 6171–6180. 10.1158/0008-5472.CAN-10-0153 20631075 PMC2912959

[B180] Sikov W. M. Berry D. A. Perou C. M. Singh B. Cirrincione C. T. Tolaney S. M.. (2015). Impact of the addition of carboplatin and/or bevacizumab to neoadjuvant once-per-week paclitaxel followed by dose-dense doxorubicin and cyclophosphamide on pathologic complete response rates in stage ii to iii triple-negative breast cancer: CALGB 40603 (Alliance). J. Clin. Oncol. 33, 13–21. 10.1200/JCO.2014.57.0572 25092775 PMC4268249

[B181] Singh H. Brave M. Beaver J. A. Cheng J. Tang S. Zahalka E.. (2017). U.S. food and drug administration approval: cabozantinib for the treatment of advanced renal cell carcinoma. Clin. Cancer Res. 23, 330–335. 10.1158/1078-0432.CCR-16-1073 27793960

[B182] Singh M. Couto S. S. Forrest W. F. Lima A. Cheng J. H. Molina R. (2012). Anti-VEGF antibody therapy does not promote metastasis in genetically engineered mouse tumour models. J. Pathol. 227, 417–430. 10.1002/path.4053 22611036

[B183] Sohn B. S. Park S. J. Kim J. E. Kim K. P. Hong Y. S. Suh C.. (2014). Single-nucleotide polymorphisms in the vascular endothelial growth factor pathway and outcomes of patients treated with first-line cytotoxic chemotherapy combined with bevacizumab for advanced colorectal cancer. Oncology 87, 280–292. 10.1159/000365593 25139485

[B184] Sorensen A. G. Emblem K. E. Polaskova P. Jennings D. Kim H. Ancukiewicz M.. (2012). Increased survival of glioblastoma patients who respond to antiangiogenic therapy with elevated blood perfusion. Cancer Res. 72, 402–407. 10.1158/0008-5472.CAN-11-2464 22127927 PMC3261301

[B185] Subbiah V. Khawaja M. R. Hong D. S. Amini B. Yungfang J. Liu H.. (2017). First-in-human trial of multikinase VEGF inhibitor regorafenib and anti-EGFR antibody cetuximab in advanced cancer patients. JCI Insight 2:90380. 10.1172/jci.insight.90380 28422758 PMC5396533

[B186] Swanton C. Govindan R. (2016). Clinical implications of genomic discoveries in Lung Cancer. N. Engl. J. Med. 374, 1864–1873. 10.1056/NEJMra1504688 27168435

[B187] Taverna G. Grizzi F. Colombo P. Graziotti P. (2013). Is angiogenesis a hallmark of prostate cancer? Front. Oncol. 3:15. 10.3389/fonc.2013.00015 23390615 PMC3565155

[B188] Teicher B. A. (1996). A systems approach to cancer therapy. (Antioncogenics + standard cytotoxics → mechanism(s) of interaction). Cancer Metast. Rev. 15, 247–272. 10.1007/BF00437479 8842498

[B189] Terme M. Pernot S. Marcheteau E. Sandoval F. Benhamouda N. Colussi O.. (2013). VEGFA-VEGFR pathway blockade inhibits tumor-induced regulatory T-cell proliferation in colorectal cancer. Cancer Res. 73, 539–549. 10.1158/0008-5472.CAN-12-2325 23108136

[B190] Thurston G. Noguera-Troise I. Yancopoulos G. D. (2007). The Delta paradox: DLL4 blockade leads to more tumour vessels but less tumour growth. Nat. Rev. Cancer 7, 327–331. 10.1038/nrc2130 17457300

[B191] Tol J. Koopman M. Cats A. Rodenburg C. J. Creemers G. J. M. Schrama J. G.. (2009). Chemotherapy, Bevacizumab, and Cetuximab in Metastatic Colorectal Cancer. N. Engl. J. Med. 360, 563–572. 10.1056/NEJMoa0808268 19196673

[B192] Tolaney S. M. Boucher Y. Duda D. G. Martin J. D. Seano G. Ancukiewicz M.. (2015). Role of Vascular Density and Normalization in Response to Neoadjuvant Bevacizumab and Chemotherapy in Breast Cancer Patients. Proc. Natl. Acad. Sci. U.S.A. 112, 14325–14330. 10.1073/pnas.1518808112 26578779 PMC4655544

[B193] Torimura T. Iwamoto H. Nakamura T. Abe M. Ikezono Y. Wada F.. (2016). Antiangiogenic and antitumor activities of aflibercept, a soluble VEGF receptor-1 and−2, in a mouse model of hepatocellular carcinoma. Neoplasia 18, 413–424. 10.1016/j.neo.2016.05.001 27435924 PMC4954942

[B194] Tyler T. (2012). Axitinib: newly approved for renal cell Carcinoma. J. Adv. Pract. Oncol. 3, 333–335. 25031963 10.6004/jadpro.2012.3.5.7 PMC4093354

[B195] Van Cutsem E. de Haas S. Kang Y.-K. Ohtsu A. Tebbutt N. C. Ming Xu J.. (2012b). Bevacizumab in combination with chemotherapy as first-line therapy in advanced Gastric Cancer: a biomarker evaluation from the AVAGAST randomized phase III trial. J. Clin. Oncol. 30, 2119–2127. 10.1200/JCO.2011.39.9824 22565005

[B196] Van Cutsem E. Tabernero J. Lakomy R. Prenen H. Prausová J. Macarulla T.. (2012a). Addition of aflibercept to fluorouracil, leucovorin, and irinotecan improves survival in a phase III randomized trial in patients with metastatic colorectal cancer previously treated with an oxaliplatin-based regimen. J. Clin. Oncol. 30, 3499–3506. 10.1200/JCO.2012.42.8201 22949147

[B197] Vasudev N. S. Reynolds A. R. (2014). Anti-angiogenic therapy for cancer: current progress, unresolved questions and future directions. Angiogenesis 17, 471–494. 10.1007/s10456-014-9420-y 24482243 PMC4061466

[B198] von Minckwitz G. Eidtmann H. Rezai M. Fasching P. A. Tesch H. Eggemann H.. (2012). Neoadjuvant Chemotherapy and Bevacizumab for HER2-Negative Breast Cancer. N. Engl. J. Med. 366, 299–309. 10.1056/NEJMoa1111065 22276820

[B199] Voron T. Colussi O. Marcheteau E. Pernot S. Nizard M. Pointet A.-L.. (2015). VEGF-A modulates expression of inhibitory checkpoints on CD8+ T cells in tumors. J. Exp. Med. 212, 139–148. 10.1084/jem.20140559 25601652 PMC4322048

[B200] Vredenburgh J. J. Desjardins A. Herndon J. E. Marcello J. Reardon D. A. Quinn J. A.. (2007). Bevacizumab plus irinotecan in recurrent glioblastoma multiforme. J. Clin. Oncol. 25, 4722–4729. 10.1200/JCO.2007.12.2440 17947719

[B201] Wang Z. Chen J.-Q. Liu J. Tian L. (2016). Exosomes in tumor microenvironment: novel transporters and biomarkers. J. Transl. Med. 14:297. 10.1186/s12967-016-1056-9 27756426 PMC5070309

[B202] Wang Z. Dabrosin C. Yin X. Fuster M. M. Arreola A. Rathmell W. K.. (2015). Broad targeting of angiogenesis for cancer prevention and therapy. Semin. Cancer Biol. 35, S224–S243. 10.1016/j.semcancer.2015.01.001 25600295 PMC4737670

[B203] Wicki A. Christofori G. (2008). The angiogenic switch in tumorigenesis, in Tumor Angiogenesis, eds Marmé D. Fusenig N. (Springer; Berlin; Heidelberg), 67–88. 10.1007/978-3-540-33177-3_4

[B204] Wilhelm S. M. Carter C. Tang L. Y. Wilkie D. McNabola A. Rong H.. (2004). BAY 43-9006 exhibits broad spectrum oral antitumor activity and targets the RAF/MEK/ERK pathway and receptor tyrosine kinases involved in tumor progression and angiogenesis. Cancer Res. 64, 7099–7109. 10.1158/0008-5472.CAN-04-1443 15466206

[B205] Wilhelm S. M. Dumas J. Adnane L. Lynch M. Carter C. A. Schütz G.. (2011). Regorafenib (BAY 73-4506): a new oral multikinase inhibitor of angiogenic, stromal and oncogenic receptor tyrosine kinases with potent preclinical antitumor activity. Int. J. Cancer 129, 245–255. 10.1002/ijc.25864 21170960

[B206] Wilke H. Muro K. Van Cutsem E. Oh S.-C. Bodoky G. Shimada Y.. (2014). Ramucirumab plus paclitaxel versus placebo plus paclitaxel in patients with previously treated advanced gastric or gastro-oesophageal junction adenocarcinoma (RAINBOW): a double-blind, randomised phase 3 trial. Lancet Oncol. 15, 1224–1235. 10.1016/S1470-2045(14)70420-6 25240821

[B207] Xu J. Wang J. Xu B. Ge H. Zhou X. Fang J.-Y. (2013). Colorectal cancer cells refractory to Anti-VEGF Treatment are vulnerable to glycolytic blockade due to persistent impairment of Mitochondria. Mol. Cancer Ther. 12, 717–724. 10.1158/1535-7163.MCT-12-1016-T 23427299

[B208] Yuan J. Zhou J. Dong Z. Tandon S. Kuk D. Panageas K. S.. (2014). Pretreatment Serum VEGF Is Associated with Clinical Response and Overall Survival in Advanced Melanoma Patients Treated with Ipilimumab. Cancer Immunol. Res. 2:127LP-132. 10.1158/2326-6066.CIR-13-0163 24778276 PMC3991109

[B209] Zhang M. Kleber S. Röhrich M. Timke C. Han N. Tuettenberg J.. (2011). Blockade of TGF-β signaling by the TGFβR-I kinase inhibitor LY2109761 enhances radiation response and prolongs survival in Glioblastoma. Cancer Res. 71:7155LP-7167. 10.1158/0008-5472.CAN-11-1212 22006998

[B210] Zhang W.-J. Li Y. Wei M.-N. Chen Y. Qiu J.-G. Jiang Q.-W.. (2017). Synergistic antitumor activity of regorafenib and lapatinib in preclinical models of human colorectal cancer. Cancer Lett. 386, 100–109. 10.1016/j.canlet.2016.11.011 27864115

[B211] Zhou Q. Guo P. Gallo J. M. (2008). Impact of angiogenesis inhibition by sunitinib on tumor distribution of temozolomide. Clin. Cancer Res. 19, 1557–1566. 10.1158/1078-0432.CCR-07-4544 18316579

[B212] Zhu A. X. Ancukiewicz M. Supko J. G. Sahani D. V. Blaszkowsky L. S. Meyerhardt J. A.. (2013). Efficacy, safety, pharmacokinetics, and biomarkers of cediranib monotherapy in advanced hepatocellular carcinoma: a phase II study. Clin. Cancer Res. 19, 1557–1566. 10.1158/1078-0432.CCR-12-3041 23362324 PMC3609423

[B213] Zhu A. X. Sahani D. V. Duda D. G. di Tomaso E. Ancukiewicz M. Catalano O. A.. (2009). Efficacy, safety, and potential biomarkers of sunitinib monotherapy in advanced hepatocellular carcinoma: a phase II study. J. Clin. Oncol. 27, 3027–3035. 10.1200/JCO.2008.20.9908 19470923 PMC2702235

